# Alternative splicing in osteoclasts and Paget’s disease of bone

**DOI:** 10.1186/s12881-014-0098-1

**Published:** 2014-08-14

**Authors:** Roscoe Klinck, Gino Laberge, Martine Bisson, Stephen McManus, Laëtitia Michou, Jacques P Brown, Sophie Roux

**Affiliations:** 1 RNomics platform, Faculty of Medicine, University of Sherbrooke, 3001, 12th Avenue North, Sherbrooke, PQ, Canada; 2 Division of Rheumatology, Department of Medicine, Faculty of Medicine, University of Sherbrooke, 3001, 12th avenue N, Sherbrooke, J1H5N4, PQ, Canada; 3 CHU de Québec, Research Centre and Division of Rheumatology, Department of Medicine, Laval University, Quebec City, PQ, Canada

**Keywords:** Alternative splicing, Osteoclast, Paget’s disease of bone, p62/SQSTM1

## Abstract

**Background:**

Mutations in the *SQSTM1/p62* gene have been reported in Paget’s disease of bone (PDB), but they are not sufficient to induce the pagetic osteoclast (OC) phenotype. We hypothesized that specific RNA isoforms of OC-related genes may contribute to the overactivity of pagetic OCs, along with other genetic predisposing factors.

**Methods:**

Alternative splicing (AS) events were studied using a PCR-based screening strategy in OC cultures from 29 patients with PDB and 26 healthy donors (HD), all genotyped for the p62^P392L^ mutation. Primer pairs targeting 5223 characterized AS events were used to analyze relative isoform ratios on pooled cDNA from samples of the four groups (PDB, PDB^P392L^, HD, HD^P392L^). Of the 1056 active AS events detected in the screening analysis, 192 were re-analyzed on non-amplified cDNA from each subject of the whole cohort.

**Results:**

This analysis led to the identification of six AS events significantly associated with PDB, but none with p62^P392L^. The corresponding genes included *LGALS8*, *RHOT1*, *CASC4*, *USP4*, *TBC1D25*, and *PIDD*. In addition, *RHOT1* and *LGALS8* genes were upregulated in pagetic OCs, as were *CASC4* and *RHOT1* genes in the presence of p62^P392L^. Finally, we showed that the proteins encoded by *LGALS8*, *RHOT1*, *USP4*, *TBC1D25*, and *PIDD* were expressed in human OCs.

**Conclusion:**

This study allowed the identification of hitherto unknown players in OC biology, and our findings of a differential AS in pagetic OCs may generate new concepts in the pathogenesis of PDB.

## Background

Osteoclasts (OCs) are multinucleated cells, whose phenotype includes the expression of the calcitonin receptor (CTR) and of the Receptor Activator of NF-κB (RANK), and the singular ability to resorb bone [1],[2]. OC formation, activity and survival are regulated by two major signaling pathways, which are activated by M-CSF and RANKL (Receptor Activator of NK-κB Ligand).

Paget’s disease of bone (PDB) is characterized by focal and disorganized increases in bone turnover, and OCs have been identified as the cells primarily affected in PDB [3]. Pagetic OCs are both larger and more numerous than those in healthy individuals. They are overactive and hypersensitive to osteoclastogenic factors [4], and are resistant to apoptosis [5]. The *SQSTM1* gene encodes the ubiquitin-binding protein sequestosome 1, also known as p62. Mutations in the *SQSTM1* gene have been identified in a high proportion of PDB patients [6], the p62P392L substitution being the most frequent [7]. In PDB, p62P392L contributes at least in part to the induction of an activated stage in OCs by stimulating signaling pathways that can lead to NF-κB activation [5],[8]. *In vivo*, knock-in mice expressing p62^P394L^ (the murine equivalent of human p62^P392L^) develop focal osteolytic lesions, some of which resemble PDB lesions [9], although in another study, co-expression of the measles virus nucleocapsid gene in the OC lineage was required [4]. Therefore *SQSTM1* mutations may not be sufficient to induce the pagetic OC phenotype, and environmental factors such as viral infection may contribute [4]. Two recently published genome-wide association studies (GWAS) in PDB patients free of *SQSTM1* mutations identified seven significant genetic variants for susceptibility to PDB located at the 1p13 *(CSF1)*, 7q33 (*CNOT4, NUP205, SLC13A4),* 8q22 (*TM7SF4), 10p13 (OPTN),* 14q32 (*RIN3),* 15q24 (*PML*, *GOLGA6A)*, and 18q21 *(TNFRSF11A)* loci [10],[11]. This implies that genes other than *SQSTM1* may also contribute to the pathogenesis of PDB, although so far only disease-causing mutations in *SQSTM1* have been identified. Besides gene expression modulation or mutations, other mechanisms regulate phenotypic variations in cells through post-transcriptional processes such as alternative splicing (AS) that leads to a vast array of transcripts with diverse functions. We hypothesized that specific RNA isoforms of OC-related genes may contribute to the pagetic OC phenotype. Our objective was therefore to look for PDB-specific AS events in these cells, and to investigate the impact of the p62^P392L^ mutation.

## Methods

### Materials

Opti Eagle’s minimum essential media (Opti-MEM), penicillin, streptomycin, fungizone, glutamine, and fetal calf serum (FCS) were purchased from Wisent (Montreal, QC). Ficoll–Paque was purchased from Amersham Biosciences (Montreal, QC). Human recombinant (hr) M-CSF, and hrGM-CSF were purchased from R&D (R&D Systems, Minneapolis, MN); soluble hrRANKL was produced in our laboratory. Rabbit polyclonal antibodies against human PIDD (#ab78389), Galectin 8 (#ab41649), and RHOT1 (#ab83779) were purchased from Abcam (Cambridge, MA); rabbit polyclonal antibodies against human TBC1D25 (OATL1) (#HPA029197), and USP4 (#U0635) were purchased from Sigma-Aldrich (St. Louis, MO), and fluorescent Alexa antibodies, Di Aminido Phenyl lndol (DAPI), and siRNA from Invitrogen (Burlington, ON).

### Clinical investigation and phenotype classification

Blood samples from each of the healthy donors (HD) and PDB patients were provided by authors JPB and LM. This research has been approved by the Ethics Committees of the CHU de Québec. All participants signed an informed-consent document before entering the study. Phenotypic expression of PDB had been previously established (JPB) in all participants based on: total serum alkaline phosphatase, total body bone scan, skull and enlarged view pelvis X-rays, and if needed, additional X-rays to confirm PDB, as described [12]. Exons 7 and 8 of the gene encoding SQSTM1/p62 had been previously sequenced in every donor and patient [13]. The p62^P392L^ mutation was the only *SQSTM1* mutation identified in individuals participating in this study. Fifty-five participants, 29 PDB (13 females, 16 males) and 26 HD (16 females, 10 males), were divided into four groups: healthy donors exempt from any known mutation in the *SQSTM1/p62* gene (HD^wt^); healthy donors carrying the p62^P392L^ mutation (HD^P392L^), these donors came from families with PDB history (carrying the p62^P392L^ mutation), but had no phenotypic expression of PDB at the time of study; PBD patients exempt from any known mutation in the *SQSTM1/p62* gene (PDB^wt^); PDB patients carrying the p62^P392L^ mutation (PDB^P392L^).

### Cell cultures

Peripheral blood mononuclear cells (PBMCs) were isolated from heparinized blood by density-gradient centrifugation, and suspended in Opti-MEM with the antibiotics, glutamine, and 2% FCS. After incubating overnight, the cells were gently washed to remove any non-adherent cells. The selected PBMCs were cultured in Opti-MEM supplemented with GM-CSF (100 pg/ml) for the first 3 days, and then for a further 3 weeks in the same medium supplemented with M-CSF (25 ng/ml) and RANKL (75 ng/ml). The medium was changed every 2–3 days. Alternatively, blood was harvested from human umbilical cord at delivery after obtaining informed consent from parturient women, as approved by our institution’s review board (University of Sherbrooke). Cord blood monocytes (CBMs) were isolated and processed as described above. These culture conditions generate multinucleated cells (MNC) that express OC markers and have the ability to resorb bone [5],[14]. Human embryonic kidney-293 T (293 T) cells were cultured in DMEM containing 10% FCS, glutamine and antibiotics.

### Alternative splicing

Total cellular RNA was extracted from fully matured OCs, reverse transcribed, and analyzed by high-throughput PCR amplification at the Université de Sherbrooke RNomics Platform as previously described [15],[16]. Five (5) ng of total RNA was used for each PCR experiment. For the detection screening we used RNA pre-amplified using a linear isothermal RNA amplification (Transplex Whole Transcriptome Amplification Kit, Sigma, Markham, ON) following the manufacturer’s protocol. AS events were characterized by end-point PCR. Primers were designed to flank the AS events, such that following amplification and analysis by microcapillary electrophoresis on Caliper LC-90 instruments (Caliper Life Sciences, Hopkinton, MA), the relative ratio of the isoforms can be deduced [15]. AS events which were amenable to characterization by high-thoughput PCR, that is, whose isoform sizes differ by between 10 and 450 nucleotides at a particular event, were selected from the RefSeq database [16]. Automated querying of this highly curated database has identified a genome-wide selection of 5223 AS events which fit the selection criteria from the full set of over 20 K human gene entries [16].

#### Detection screen

In the first step of the screen, termed the “detection screen”, primer pairs targeting the 5223 AS events were used to perform PCR and microcapillary analysis on pre-amplified RNA in representative samples of the four groups, each sample being a pooled RNA from five patients (60 ng of total RNA per individual needed prior to pre-amplification). The purpose of this screen was to detect AS events in the sample pools for which both isoforms were detectable to a minimum of 10% of the total signal in at least one of the samples. Such AS events are designated as “active”. If only one form of the event is detected in all samples, or if no amplification of the expected products occurs, these AS events were not considered for subsequent validation.

#### Validation screen

Next, during what we term the “validation screen”, we analyzed the active AS events in PCR experiments on non-amplified RNA in our cohort of 55 individuals, using the same primers, however in this screen, the relative amounts of long and short isoforms issued from the microcapillary analysis of the amplification reaction was considered. The splicing profile from each PCR reaction was expressed as a percent splicing index (PSI or Ψ) which is the ratio of the long isoform concentration to the total (long + short) isoform concentrations, expressed as a percentage. The Ψ values of the active AS events were compared in the four groups. The use of multiple samples reduces the impact of individual aberrant AS, artefacts related to the pre-amplification procedure, and to confirm the PDB-specific events.

### Gene expression study

For the quantitative real-time RT-PCR analysis, total RNA was extracted from fully matured OCs using the RNeasy plus kit (Qiagen, Mississauga, Canada), including a DNase digestion step. At least 50 μg of RNA was harvested from each cell sample. After being assayed and checked for quality, 1 μg of total RNA was reverse transcribed using the high-efficiency QuantiTect Primer Assay kit (Qiagen), and sent for quantitative PCR amplification (RNomics Platform, University of Sherbrooke). Human primers of candidate genes were generated and validated, and the real-time PCR reaction was conducted with 200 nM of primers, 10 ng of cDNA and 5 μl of Power SYBR-green Master Mix in a total volume of 10 μl. Amplification and detection of the candidate genes and of three reference genes (Splicing factor 3a, subunit 1 (*SF3A1*), Proteasome 26S subunit ATPase 4 (*PSMC4*), Pumilio homolog 1 (*PUM1*)) were conducted with a Realplex 2 Master Cycler (Eppendorf, Mississauga, Canada).

We investigated the relative expression of genes for which AS events were significantly associated with PDB in OC cultures. All the primers were designed based on sequences reported in the Aceview database [17]. Three primer pairs were designed for each gene, in order to choose the best primer pair after validation. All primers pairs were validated by qPCR amplification of a total XPressRef Universal human mRNA (Cedarlane, Burlington, ON). Amplification products were submitted to capillary electrophoresis in order to validate the size of the expected product. In addition, a melting curve was generated and only those primers giving one product were selected. If all the three primer pairs gave such results, the pair giving the lowest Ct was selected. Finally, the primer pair efficiency was obtained from a standard curve experiment where a series of dilution of the same sample was correlated to the Ct values.

All samples were run in triplicate for the target and reference genes. Relative expression levels were normalized with respect to a set of reference primer pairs (i.e. the geometric mean of the three reference genes). Relative expression quantification analysis relied on the qBase method [18]. This method constitutes an improvement over the classical delta-delta-Ct method, which was extended to take into account multiple stably expressed reference genes for improved normalization.

### RNA inactivation

Seventy-two to 96 hours before lysing cells, 293 T cells were transfected either with a specific siRNA (50 nM), or with a negative control siRNA (scrambled siRNA) in a solution containing Lipofectamine 2000 Transfection Reagent in serum-free Opti-MEM. One specific siRNA was used to inhibit each gene (*PIDD, TBC1D25, RHOT1, LGALS8*), two siRNAs against *USP4* were used in combination (Table 1). The cells were incubated at 37°C for 2 hours. The subsequent downregulation of all targeted proteins was assessed by western-blotting.

**Table 1:** siRNA sequences

**Gene symbol**	**siRNA Sequence**
PIDD	CACCGGAGGGGACACUGCUdTdT
TBC1D25	UCAUCCGAGCCUUUGAUUUdTdT
RHOT1	ACUUGUUGUUGCAUGAUAUdTdT
USP4 (1)	CAGGUUGAGGAAUGAUUCUdTdT
USP4 (2)	GAGGAAUGAUUCUGUGAUUdTdT
LGALS8	CAAUCCAGGUAACCUUUAAdTdT

### Western-blotting

Cells were cultured as described above. Proteins were extracted and lysates were quantified by modified Bradford assay (Bio Rad, Mississauga, ON) and run through SDS-PAGE. Western-blots were performed by incubating a primary antibody against Galectin 8, RHOT1, USP4, TBC1D25, PIDD, or β-actin where appropriate overnight at 4°C. HRP-conjugated secondary antibodies were used to achieve detection with a chemiluminescent system.

### Immunofluorescence

At the end of the OC cultures, cells were washed quickly with cold PBS, fixed with 1% para-formaldehyde, and then permeabilized in PBS-Triton X-100 (0.2%). Specific antibodies directed against USP4, TBC1D25, and PIDD were incubated in Antibody diluent (DAKO) overnight at 4°C. Alexa-488 (green) anti-rabbit antibodies were incubated for 2 h at room temperature, along with Alexa Fluor 633 phalloidin. DAPI counter-staining was performed, so that OCs containing more than three nuclei could be visualized. Sequences of pictures were taken with appropriate filters to show all three colors, and superimposed with Simple PCI software.

### Statistical analysis

We studied four groups (PDB^wt^, PDB^P392L^, HD^wt^, HD^P392L^) in six intergroup analyses (all PDB *vs* all HD; all PDB patients *vs* HD^wt^; PDB^wt^*vs* HD^wt^, PDB^wt^*vs* HD^P392L^, PDB^wt^*vs* PDB^P392L^; and HD^wt^*vs* HD^P392L^). Student’s t-tests were applied to the Ψ values for each AS event in each group and Ψ value changes with values of p < 0.05 were considered to be significant, with a correction for multiple testing using the false discovery rate or q-value [19]. For genes in which we confirmed the presence of specific AS events of PDB OCs, we looked for association with the *P392L* mutation using Fisher exact tests. For gene expression analysis, the fold change was calculated as the ratio of the mean relative expression for each group, and results were expressed as the base 2 logarithm of the fold change. Statistical significance was defined as p < 0.05 using a Student’s t-test.

## Results

### Alternative splicing in Paget’s disease of bone

To perform our study of AS in PDB, we used a validated model for the *in-vitro* study of human OCs involving peripheral blood mononuclear cells (PBMCs) [5]. We studied OC cultures from a cohort of patients (PDB) and healthy donors (HD), all genotyped for the p62^P392L^ mutation, and representing four distinct groups: HD^wt^ (n = 16, mean age 61 years; 39–84), HD^P392L^ (n = 10, mean age 51.3 years; 33–70; of which three were under 52 years old), PDB^wt^ (n = 17, mean age 70.7 years; 45–86), PDB^P392L^ (n = 12, mean age 75 years; 59–85). In the first step of the AS screen, termed the “detection screen”, primer pairs targeting 5223 characterized AS events were used to amplify and evaluate the relative isoform ratios on pooled cDNA from our four groups (5 patients in each group). 1056 of the targeted AS events showed evidence of regulation, and were thus classified as “active”. Three genes of the 5223 tested AS events (*VDR*, *OPTN*, and *PML*) had been previously associated with PDB [6],[11], of which *OPTN* and *PML* displayed active AS events.

From this active list (n = 1056), 192 AS events (in 164 genes) were selected based on genes that have a confirmed or potential function in the OC phenotype, function or signaling pathways, or those whose involvement in PDB has been suggested [5],[10],[20] (Additional file 1: Table S1). The 192 active AS events were then tested in a “validation screen”. Five individuals from each group were analyzed separately using the computed Ψ values (see Methods) to compare profiles between groups. This screen yielded 21 AS events that were either significantly associated with PDB or with the p62^P392L^ mutation (t-test, p < 0.05, Additional file 2: Table S2). We then confirmed this association by analyzing these 21 active AS events by PCR on cDNA from the remaining non-tested individuals (12 PDB^wt^, 7 PBD^P392L^, 11 HD^wt^, 5 HD^P392L^), and Ψ values were compared (Table 2).

**Table 2:** Validation of the selected 21 alternative splicing events in the whole cohort

**Gene**	**Forward Primer**	**Reverse Primer**	**all PDB** ***vs*** **HD**		**all PDB** ***vs*** **HD**^**wt**^		**PDB**^**wt**^ ***vs*** **HD**^**wt**^		**PDB**^**wt**^ ***vs*** **HD**^**P392L**^		**PDB**^**wt**^ ***vs*** **PDB**^**P392L**^		**HD**^**wt**^ ***vs*** **HD**^**P392L**^	
			***(n =19 vs 16)***		***(n =19 vs 11)***		***(n =12 vs 11)***		***(n =12 vs 5)***		***(n =12 vs 7)***		***(n =11 vs 5)***	
			p*	***q***	***p***	***q***	***p***	***q***	***p***	***q***	***p***	***q***	***p***	***q***
***LGALS8***	**ACACTCTGGGCATTTATGGC**	**TTTAACGACGACAGTTCGTCC**	*0.00001*	0.0002	*0.0005*	0.009	*0.0004*	0.004	*0.001*	0.008	0.64	0.55	0.71	0.55
***USP4***	**AGCTATTCAACATCCCTGCG**	**GGTGGCTCCTGACAATTATACG**	*0.0002*	0.0012	*0.0019*	0.013	*0.002*	0.006	*0.033*	0.054	0.73	0.55	0.48	0.51
***CASC4***	**GAGGTGGTGATGCAGGGATGC**	**GATCCATTTGAAGCTCTCGTTCTTC**	*0.001*	0.004	*0.001*	0.009	*0.001*	0.004	*0.050*	0.07	0.15	0.27	0.22	0.39
***RHOT1***	**TCCACCACAAGCCTTCACTT**	**GTGCACATACAGCGTAACCG**	*0.001*	0.004	*0.018*	0.061	*0.021*	0.042	*0.003*	0.0019	0.88	0.59	0.89	0.66
***PIDD***	**ATGTTCGAGGGCGAAGAGTT**	**CGCCACGGTAGAAGGACAC**	*0.01*	0.03	*0.03*	0.08	*0.03*	0.04	*0.03*	0.05	0.68	0.55	0.62	0.51
***LGALS9***	**GTGATGGTGAACGGGATCCT**	**GTTGGCAGGCCACACGCC**	*0.02*	0.03	0.053	0.11	0.14	0.18	0.10	0.12	0.38	0.42	0.81	0.68
***TBC1D25***	**AATGTGAGAGCTTCTTGCCG**	**AGGATGGACTGTGTAAAGGGC**	*0.03*	0.05	*0.04*	0.10	*0.01*	0.02	*0.02*	0.05	*0.04*	0.24	0.35	0.39
***THAP1***	**AGGACAAGCCCGTTTCTTTC**	**TCCAATAGCAGCATCAACCTG**	*0.04*	0.09	*0.01*	0.051	*0.01*	0.04	0.43	0.44	0.53	0.49	0.27	0.39
***ABTB1***	**GAGCAGCGAGACGTGGAG**	**GCTTGTAATCGCGTAGAGCC**	0.09	0.33	*0.01*	0.057	*0.02*	0.08	0.56	0.44	0.12	0.27	0.08	0.39
***WARS***	**AGCGTGACCAGTGGCCAC**	**GCCTTTTGCACTGCTTGTCT**	0.28	0.39	0.51	0.67	0.84	0.85	0.48	0.44	0.15	0.27	0.50	0.55
***PIDD***	**CTGAGCTTGGACCTGTACCC**	**AGGCCACTCAGACCAGCG**	0.23	0.39	0.42	0.65	0.47	0.59	0.73	0.47	*0.03*	0.24	0.27	0.39
***METTL13***	**CATGCGTGCGTTTGTCGT**	**ATGTCGATGTTCACTATATCCCG**	0.42	0.56	0.78	0.87	0.50	0.50	0.01	0.05	0.11	0.27	0.29	0.39
***L3MBTL***	**CCAAGTGGACCATCGATGAG**	**AGCAAATGAGTGGGTAGAGAGC**	0.34	0.56	0.22	0.43	0.24	0.49	1.00	0.56	0.82	0.59	0.22	0.39
***BTC***	**ACCACCACACAATCAAAGCG**	**TTACGACGTTTCCGAAGAGG**	0.41	0.56	0.57	0.70	0.80	0.79	0.75	0.47	0.19	0.29	0.51	0.51
***NLRP12***	**TCAGTGTGAACCAGAGCCTG**	**CAGGTAAAGGTCGGTCAAGG**	0.41	0.56	0.80	0.87	0.38	0.50	0.03	0.05	0.22	0.29	0.25	0.39
***CASC4***	**GCTCAAACTTGGACAGTGAACC**	**GCAGGATCCATTTGAAGCTC**	0.66	0.92	0.42	0.65	0.24	0.48	0.87	0.52	0.37	0.42	0.21	0.39
***ABI1***	**AGTTCTGGATGATGTGGGCC**	**TGGCGGTTTCTGAGTAGGAGGA**	0.85	0.94	0.46	0.65	0.37	0.50	0.52	0.44	0.48	0.49	0.20	0.39
***RAB3IP***	**TGGGATTACAACCTATCCGG**	**CCCTGCTGAATGTATCGAATG**	0.89	0.94	0.44	0.65	0.50	0.53	0.44	0.44	0.98	0.63	0.24	0.39
***PRMT2***	**GCTCCTGGAAAGGACCGTC**	**TTAGGCTCTGCCTCATAACTGC**	0.85	0.97	0.93	0.87	0.93	0.85	0.70	0.47	n/a	n/a	0.28	0.39
***BCL2L12***	**TCCTAGCTGCCTTCCTTAG**	**GTATGGCTTCCTTCTCTGTC**	0.87	0.92	0.86	0.87	0.48	0.61	0.58	0.44	0.14	0.27	0.95	0.68
***BCL2L15***	**TTGAGGAACAAACGGAATGC**	**AGCTAAGCTGGAATCCTGAGC**	0.98	0.98	0.91	0.87	0.87	0.82	n/a	n/a	n/a	n/a	n/a	n/a

*Student’s t-test of Ψ (PSI) values for each AS event in each group (p-value) with a correction for multiple testing using the false discovery rate (q-value).

HD^wt^ healthy donors exempt from *SQSTM1/p62* mutation (n=11); HD^P392L^ healthy donors carrying p62^P392L^ (n=5); PBD (Paget’s disease of bone); PBD^wt^ patients exempt from *SQSTM1/p62* mutation (n=12); PDB^P392L^ patients carrying the p62^P392L^ (n=7). n/a: missing data (inadequate RNA samples).

This led to the identification of six AS events that were significantly associated with PDB, corresponding to: *LGALS8* (galectin-8), *RHOT1* (mitochondrial Rho GTPase1), *CASC4* (cancer susceptibility candidate 4), *USP4* (ubiquitin specific protease 4), *TBC1D25* (TBC1 domain family, member 25), and *PIDD* (p53-induced death domain protein) (Table 3). No significant association of AS profile with the p62^P392L^ mutation was found.

**Table 3:** Selection of six genes whose alternative splicing is associated with Paget’s disease of bone (PDB)

	**Description**			**Ψ values (long/long +short) (+/− SD)**		**all PDB** ***vs*** **HD**
**Gene symbol**	**Protein name**	**AS event description**	Alternative products*	**All PDB**	**All HD**	p-value**
				**n=19**	**n=16**	
***LGALS8***	lectin, galactoside-binding, soluble, 8 Galectin-8	cassette exon, 142 nt, coding region, in frame	- isoform 1: 317 aa, 36 kDa (short)	0.08 (0.04)	0.14 (0.04)	0.00001
			- isoform 2: 359 aa, 40.5 kDa (long)			
***USP4***	ubiquitin specific peptidase 4	cassette exon, 141 nt, coding region, in frame	- isoform 1: 963 aa, 109 kDa (long)	0.20 (0.04)	0.27 (0.06)	0.0002
	(proto-oncogene)		- isoform 2: 916 aa, 104 kDa (short)			
***CASC4***	cancer susceptibility candidate 4	cassette exon, 168 nt, coding region, in frame	- isoform 1: 436 aa, 49 kDa (long)	0.21 (0.06)	0.29 (0.06)	0.001
			- isoform 2: 380 aa, 43 kDa (short)			
			- isoform 3: 177 aa, 21 kDa			
***RHOT1***	ras homolog family member T1	cassette exon, 96 nt, coding region, in frame	- isoform 1: 618 aa, 71 kDa	0.16 (0.04)	0.23 (0.08)	0.001
			- isoform 2: 659 aa, 75 kDa (short)			
			- isoform 3: 691 aa, 80 kDa (long)			
	MIRO1		- isoform 4: 580 aa, 66 kDa			
***PIDD***	p53-induced death domain protein	3′ AS site, 51 nt, coding region, in frame	- isoform 1 910 aa, 100 kDa (long)	0.63 (0.06)	0.58 (0.05)	0.01
			- isoform 2: 893 aa, 98 kD (short)			
			- isoform 3: 753 aa, 83 kDa			
	LRDD		- isoform 4: 597 aa, 67 kDa			
***TBC1D25***	TBC1 domain family, member 25	cassette exon, 155 nt, 5′ untranslated region	- isoform 1: 688 aa, 76 kDa (long)	0.63 (0.05)	0.68 (0.06)	0.03
	OATL1		- isoform 2: 100 aa, 10 kDa (short)			

*The “long” and “short” isoforms correspond to the “long” and “short” mRNA variants we investigated.

PBD (Paget’s disease of bone) (n=19); HD healthy donors (16).

**The Ψ (PSI) values for each AS event have been t-tested (p-value) (all PBD vs HD).

### Expression of genes with PDB-related AS events in OCs

**Figure 1:**
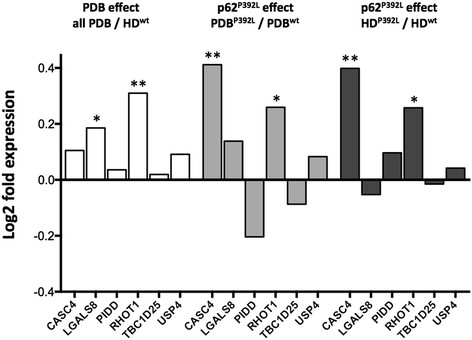
**Gene expression profile of the six selected genes.** We investigated the relative gene expression profile of the six genes with an AS event significantly associated with PDB (*CASC4, LGALS8, PIDD, RHOT1, TBC1D25, USP4*) in OC cultures from 22 PDB patients (including 10 PDB^P392L^) and 22 healthy donors (HD) (including 10 HD^P392L^). Total RNA extraction was performed on cultures from PBMC-derived OCs, followed by real-time PCR experiments. Relative levels were normalized with respect to a set of three reference genes. We compared the mean, normalized, relative expression between the four groups using Student’s t-test. Differential expression is reported as the log2 ratio. Comparisons between all PBD and HD^wt^, PBD^P392L^ and PBD^wt^ as well as HD^P392L^ and HD^wt^ are presented. *p < 0.05, **p < 0.01.

The OC gene expression profiles of the six genes *LGALS8*, *RHOT1*, *CASC4*, *USP4*, *TBC1D25*, and *PIDD,* were analyzed in the four groups (12 PDB^wt^, 10 PBD^P392L^, 12 HD^wt^, 10 HD^P392L^), and the results in PBD patients relative to healthy donors, in presence of the p62^P392L^ mutation or not, are shown in Figure 1. In the comparison between the gene expression (mean +/− SEM) in all PDB patients regardless of whether the p62^P392L^ mutation was present or not *versus* non-mutated healthy donors, there was a significant up-regulation in the *RHOT1* gene (1.06 ± 0.04 in PBD *vs* 0.85 ± 0.03 in HD^wt^, *p* =0.002), and to a lesser extent in the *LGALS8* gene (1.07 ± 0.03 *vs* 0.94 ± 0.03, *p* =0.015) in PDB patients. When comparing the relative gene expression between OCs from PDB patients carrying p62^P392L^ and PDB patients with no mutation in the *SQSTM1* gene, we observed a significant increase in the expression of the *CASC4* gene in presence of the mutation (1.34 ± 0.09 in PDB^P392L^*vs* 1.01 ± 0.04 in PDB^wt^, *p* =0.002), and in the expression of *RHOT1* (1.17 ± 0.06 *vs* 0.97 ± 0.04, *p* =0.021). Finally, the relative expression of two genes was significantly upregulated in OC cultures from HD carriers of p62^P392L^ compared to healthy donors with no mutation in the *SQSTM1* gene, with an upregulation of *CASC4* (1.39 ± 0.40 in HD^P392L^*vs* 1.05 ± 0.11 in HD^wt^, *p* = 0.015), and *RHOT1* genes (1.02 ± 0.06 in HD^P392L^*vs* 0.85 ± 0.06 in HD^wt^, *p* = 0.026) (Figure 1).

### Protein expression of *PIDD, TBC1D25, RHOT1, LGALS8* and *USP4* in mature OCs

A major interest of this study is that it enabled us to identify AS events from genes not previously known to be associated with OC biology. We therefore studied the expression of these genes in human OCs at the protein level, focusing on five genes (*PIDD*, *TBC1D25*, *RHOT1, LGALS8* and *USP4*), as they were previously reported for their involvement in NF-κB signaling, apoptosis, autophagy, and ubiquitination in other systems, which are collectively recognized as pathways essential to OC biology [21]. As we were unable to find functioning antibodies against CASC4, it was excluded from further study. To evaluate the expression of the encoded proteins, we used OCs derived from cord blood monocytes (CBMs), and investigated the protein-level expression by immunofluorescence and western blot using specific antibodies. Our objective was to confirm the expression of the proteins encoded by the selected genes in human OCs, for which the CBM-derived OCs represent a reliable model [8],[14],[22].

**Figure 2:**
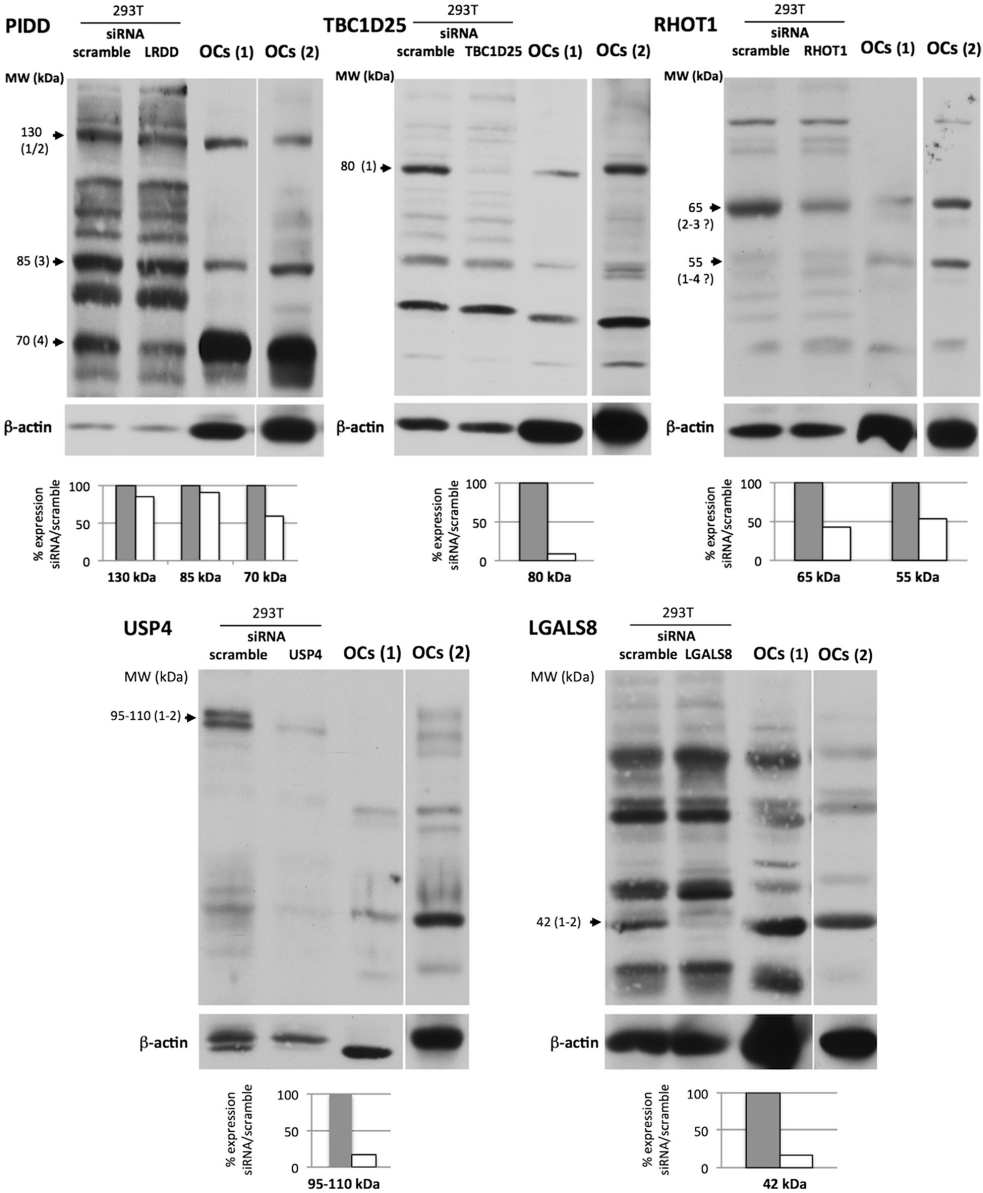
**Western blot analysis of** ***PIDD*,** ***TBC1D25*,** ***RHOT1*,** ***USP4*** **and** ***LGALS8*** **encoded proteins.** The expression of PIDD, TBC1D25, RHOT1, USP4 and LGALS8 was analyzed by western blot of a protein extract of OC cultures derived from CBMs, and from 293 T cells. β-actin was used as an internal control. 293 T cells were transfected with a scrambled control siRNA (scramble), or with an siRNA specific for each gene (regions common to all isoforms were targeted). Western blot analyses of each encoded protein were performed 72 to 96 hours post siRNA transfection in 293 T cultures, and in OC cultures, with antibodies directed against PIDD, TBC1D25, RHOT1, USP4 and LGALS8 proteins or actin. Images are representative of three independent experiments. Two different gels are shown for the analysis in OCs. Putative isoforms are indicated to the left of each gel image (numbering from Table 3).

Western blotting showed that all the proteins encoded by the *PIDD*, *TBC1D25*, *RHOT1, USP4,* and *LGALS8* genes were indeed expressed in human OC cultures (Figure 2). To identify and validate some of the isoforms of the candidate proteins observed in OCs, we also analyzed the effects of siRNA-mediated depletion of these genes (Table 1) at the protein level. 293 T cells were used for this as OCs cannot be transfected so efficiently. The antibodies used were all directed against a peptide shared by all the described isoforms for each of the proteins, except for anti-TBC1D25 antibodies, which are directed against a peptide within isoform 1 but containing only 25% of the isoform 2 sequence. PIDD isoform 4 (70 kDa) was the main isoform expressed by OCs, and its expression was confirmed by siRNA knockdown. Two other bands of higher MW (130 kDa and 85 kDa) were also detected in OCs and may correspond to the other isoforms (1/2 and 3, respectively), but were not clearly down-regulated by siRNA. The expression of the long isoform 1 of TBC1D25 was specifically detected (80 kDa). Two isoforms of RHOT1 were primarily detected in OCs (55 and 65 kDa), but we were not able to differentiate between specific isoforms as the observed bands migrated faster than predicted. Two proteins of high MW (95 and 110 kDa) were detected in the USP4 analysis, which may correspond to the two known isoforms. Only one Galectin 8 isoform of 42 kDa was specifically detected (Figure 2).

**Figure 3:**
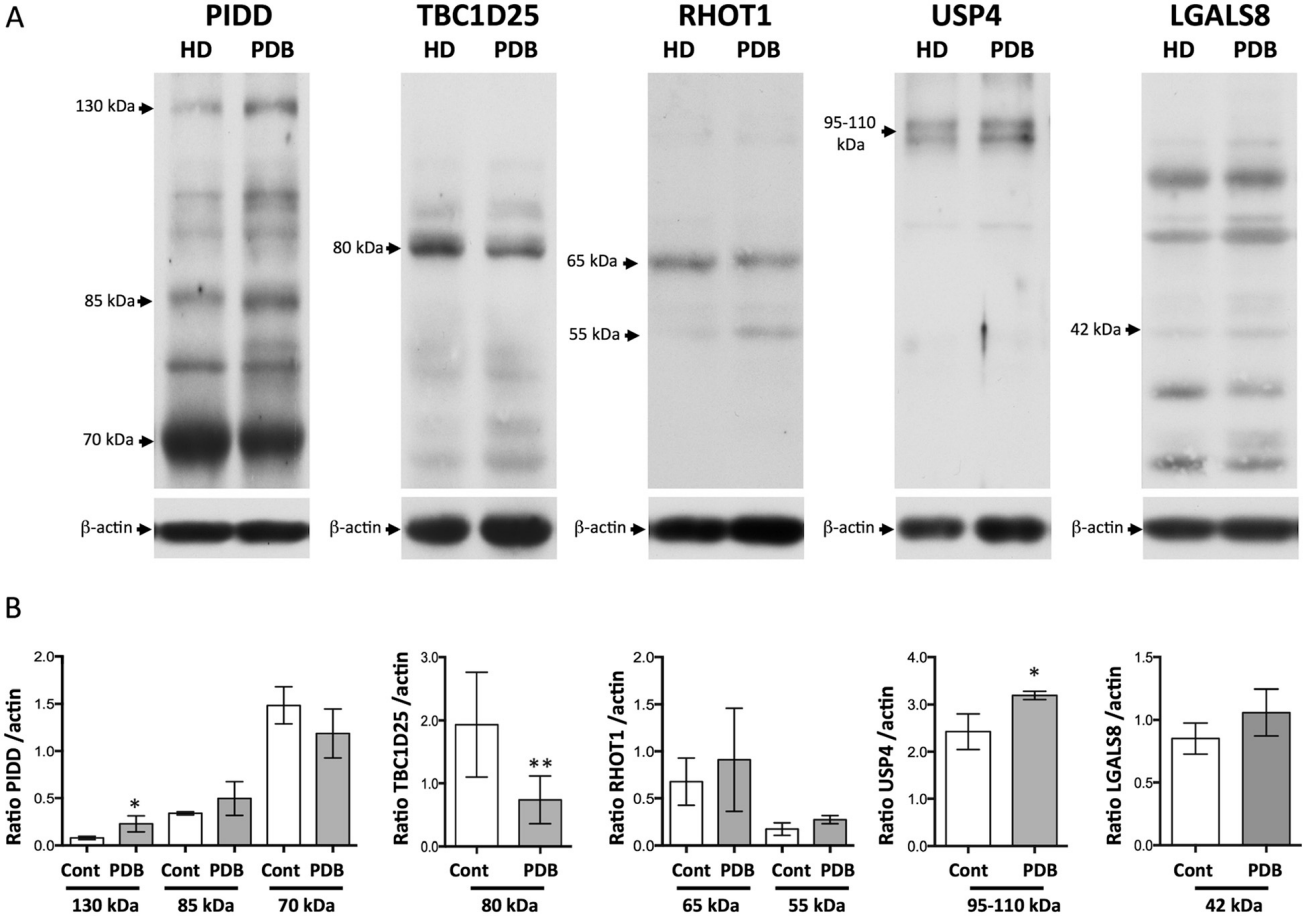
**Western blot analysis of** ***PIDD*,** ***TBC1D25*,** ***RHOT1*,** ***USP4*** **and** ***LGALS8*** **encoded proteins in pagetic or control OCs.** The protein expression of the candidate genes was analyzed in OC cultures derived from PDB or HD without any p62 mutations. **A**- Western blot analyses of each encoded protein were performed using antibodies directed against PIDD, TBC1D25, RHOT1, USP4 and LGALS8 proteins or actin. **B**- Optical densities for bands corresponding to each protein were corrected with the optical density obtained for bands corresponding to actin, and computed in graphical representations (n = 4 to 6 in each group, in 2 independant experiments). Analyses are reported as mean ratio ± SD (*p ≤ 0.05, ** p ≤ 0.01).

The same protein profile was observed in OC cultures from non-mutated PDB or HD, and we were not able to quantify the expression of the short and long isoforms (as defined in Table 3), as the long and short isoforms of four candidate proteins (PIDD, RHOT1, USP4, and galectin-8) showed similar migration rates. The relative expression of TBC1D25 long isoform 1 (the short isoform being non-detectable) was significantly lower in PDB OCs compared to HD OCs (*p* =0.009) (Figure 3).

**Figure 4:**
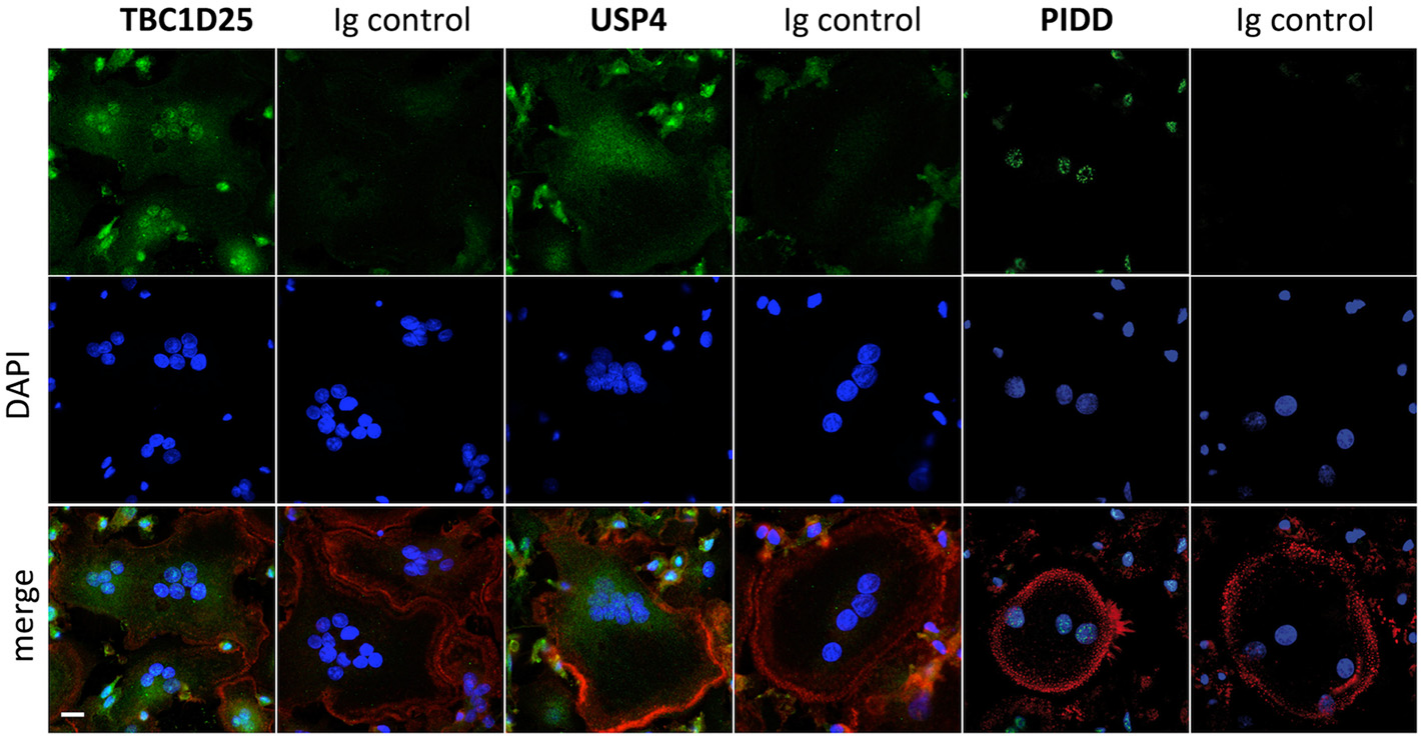
**Immunofluorescent analysis of** ***TBC1D25*,** ***USP4*** **and** ***PIDD*** **encoded proteins in mature human OCs.** Immunofluorescence tests were performed on mature osteoclasts derived from CBMs, using antibodies directed against each protein (encoded by *TBC1D25*, *USP4* and *PIDD*), and fluorescent secondary antibodies as well as Alexa Fluor 633-conjugated phalloidin. Nuclei were stained with DAPI. Images are representative of three independent experiments. Scale bar represents 10 μm.

In order to compensate for effects potentially attributable to non-osteoclastic cells included in the populations used for western blotting, we included single-cell analysis using immunofluorescence to evaluate the expression of the *PIDD*, *TBC1D25*, and *USP4* encoded proteins in human OCs. We found that mature multinucleated OCs expressed PIDD, predominantly in the nucleus. The expression of TBC1D25 and USP4 was cytoplasmic in OCs (Figure 4).

## Discussion

AS plays a central role in protein diversity and post-transcriptional gene regulation. The role of AS in the production of oncogenes and tumor suppressors is of major interest, as variants of these genes are often found specifically in tumors from diverse tissues [23],[24]. In non-neoplastic diseases, the functional importance of AS is best demonstrated by its capacity to cause diseases such as muscular dystrophies and premature-aging disorders [25]. In spite of its imposing biological relevance, little is known about the effects of AS isoforms in bone diseases. Splicing site *SQSTM1* mutations in PDB have been reported at least twice [26],[27]. Alternative splicing mutations have also been reported in other bone diseases, such as in TCIRG1-linked autosomal recessive osteopetrosis [28], however the present study is the first to investigate AS in OCs in PDB. A major strength of our approach lies in using human OCs derived from circulating OC precursors from PBD patients and healthy controls. Culture of OCs in vitro may help to identify the genetic contribution that may cause intrinsic alterations in OCs, by comparison with appropriate normal donors. We hypothesized that specific RNA splicing of OC-related genes may contribute to the OC phenotype and the clinical expression of PDB. Using a screening strategy that has been applied to cancer research in our institution [16], we found AS events significantly associated with PDB, but not with p62^P392L^, in six genes: *LGALS8*, *RHOT1*, *CASC4*, *USP4*, *TBC1D25*, and *PIDD*. While gene selection biases may have been present in both the detection and validation screens, as, for economic reasons, not all known AS events were screened, the identification of the six candidate genes from this small subset underscores potentially significant role of AS in the development and progression of PDB. Upregulation of *LGALS8* and *RHOT1* genes was observed in pagetic OCs, and the p62^P392L^ mutation was associated with an upregulation of *RHOT1* and *CASC4*. The encoded proteins were all detected in immunoblotting analyses in OC cultures, and three of them (PIDD, TBC1D25 and USP4) were expressed in human mature OCs. While not all isoforms have been clearly discriminated by western blot analysis, thus limiting our capacity to examine isoform ratios between PBD and HD at the protein level, subtle changes in the splice-isoform ratios might be associated with disease susceptibility [29]. In addition to providing evidence of an AS modulation in PDB, our findings also enabled us to identify AS events from genes not previously known to be associated with OC biology.

In OCs, the p62 scaffolding protein is one of the functional links reported between RANKL and TNFR-associated factor 6 (TRAF6)-mediated NF-κB activation [21],[30]. Protein p62 promotes the binding of CYLD to TRAF6 [31], a de-ubiquitinating enzyme (DUB) that negatively regulates NF-κB activity by reducing TRAF6 auto-ubiquitination [32]. The p62^P392L^ mutation abolished CYLD interaction and enhanced OC formation and activity [33]. USP4 (Ubiquitin-specific peptidase 4) is another DUB, of which two isoforms are produced by AS in humans [34]. Its functional properties include the de-ubiquitination of TAK1 [35], an important kinase in the RANKL-induced signaling complex [36]. In HEK293 cells, USP4 has also been shown to associate with TRAF6 and to inhibit TRAF6 ubiquitination, and consequently NF-*κ*B activation [37]. USP4 could therefore play a role in the p62/TRAF6-related signaling in pagetic OCs.

Pagetic OCs are overactive and resistant to apoptosis, a phenotype partly related to p62^P392L^[5]. We found a differential splicing in RNA of genes involved in apoptosis (*CASC4, RHOT1, PIDD*). The increased expression of *CASC4* has been associated with HER-2/neu proto-oncogene overexpression, a membrane tyrosine kinase linked to cell proliferation and survival pathways [38]. RHOT1, also named MIRO-1, is a Rho GTPase involved in mitochondrial transport. Overexpression of its constitutively active mutant resulted in an increased apoptotic rate [39]. *LGALS8* encodes Galectin-8, a beta-galactoside-binding lectin which has been implicated in cell spreading [40],[41], and in the modulation of neutrophil functions [42]. Galectin-8 also appears as a potent pro-apoptotic agent in T cells and inflammatory cells [43],[44], and is a strong inducer of ERK activation [41],[44]. In addition to USP4 that may modulate p53 degradation, PIDD, also named LRDD (leucine-rich repeats and death domain containing), is an effector of p53-induced apoptosis. PIDD is able to induce NF-κB activation [45], but is also able to form a caspase- 2-activating platform to cause cell death [46]. PIDD isoform 1 is the only isoform able to activate caspase-2, whereas isoforms −1, −2, −3 activate NF-κB [47]. Isoform 4 is regulated by p53 and when overexpressed independently promotes apoptosis [48]. While isoform 4 did not show significant differences between PDB and non-PDB samples, and isoform 2 did not pass the detection screen selection criteria, we found a slight decrease in isoform 1 relative to isoform 3 in PDB samples, implying a more anti-apoptotic behaviour of PIDD in PDB than non-PDB samples. None of these genes (*PIDD, RHOT1, LGALS8*) has ever been associated with PDB, and all represent good candidates for further investigation of the OC survival in PDB.

A pathognomonic feature found in PDB patients is the presence of inclusion bodies in OCs within affected bone; because these inclusion bodies resemble the p62-aggregates observed in diseases involving defective autophagy, dysregulation of the autophagy process may well be part of the pathogenesis of PDB [49]. A dysregulation of the autophagy process has been suggested in OCs from knock-in mice expressing p62^P394L^[9], and an impaired autophagosomal degradation of p62 has been observed in HEK 293 cell lines transfected with PDB-related p62 variants [50]. OATL1/TBC1D25*,* a Rab-GTPase-activating protein (Rab-GAP), of which two spliced isoforms exist, is a newly-discovered binding partner of Atg8/LC3, and has been shown to be involved in a late stage of autophagosome maturation in MEFs and COS-7 cells [51]. Our results have identified TBC1D25 as a potential candidate that may be worth investigating in PDB.

A limitation of our study is that expression of the identified genes and their RNA isoforms occurred in cells that were analyzed in long-term *in-vitro* cultures. These conditions may have been subject to stress-induced aberrant gene expression, such as cancer susceptibility *CASC4*, or genes related to p53 which is induced by DNA damage or stress stimuli such as *PIDD,* and *USP4*. Yet, the proteins encoded by these genes were found to be expressed in human OCs, and their splicing profiles were significantly modified in OCs from PBD patients compared to controls. In addition, except for the PIDD isoforms, the functional impact of the different splice variants of the six genes reported here remain elusive.

## Conclusion

Our screening strategy led to the identification of AS events that were significantly associated with pagetic OCs, and our findings allowed the identification of hitherto unknown players in OC biology. Further studies are warranted to identify the exact function of the *LGALS8*, *RHOT1*, *CASC4*, *USP4*, *TBC1D25*, and *PIDD* genes in human OCs, to investigate the regulation and functional impact of the identified specific isoforms in OCs using isoform-specific inhibitors, and their respective contribution to the OC phenotype in PDB. Our results may identify future avenues for research, as AS modulation and its transposition to OC biology represent a new area of research with wide-ranging potential interest for explaining how these cells are regulated. This new approach may also generate new concepts in the pathogenesis of Paget’s disease.

## Competing interests

All authors confirm that they have no competing interest.

## Authors’ contributions

RK and SR devised the study and analyzed the data. RK conducted the splicing and gene expression studies. MB, GL and SM carried out cell cultures and immunoblots. GL carried out immunofluorescence studies. LM and JPB provided blood samples from each of the healthy donors and PDB patients. SR drafted the paper. All authors reviewed the data, revised the manuscript and approved the final manuscript.

## Additional files

## Supplementary Material

Additional file 1: Table S1. Selection of 164 genes for the validation study. Click here for file

Additional file 2: Table S2. First validation study. Click here for file
