# Supplementary material for: Alternative splicing in osteoclasts and Paget’s disease of bone
Source: BMC Med Genet. 2014 Aug 14;15:98. doi: 10.1186/s12881-014-0098-1 (PMC4143580; doi:10.1186/s12881-014-0098-1)
Supplement: Additional file 1: Table S1. — Selection of 164 genes for the validation study. [file s12881-014-0098-1-S1.pdf]

Supplemental Table 1: Selection of 164 genes for the validation study

Genes (164)	Full gene name approved by the HGNC; synonym	Pathways (non exhaustive)
ABI1	abl-interactor 1	Transduction of signals from Ras to Rac-cytoskeleton
ABTB1	ankyrin repeat and BTB (POZ) domain containing 1	PTEN signaling pathway
AKT2	protein kinase Akt-2	Akt pathway
ANXA6	annexin A6	Calcium-dependent membrane and phospholipid binding proteins
APAF1	apoptotic peptidase activating factor 1	Apoptosis
APC	Adenomatous Polyposis Coli	Antagonist of the Wnt signaling pathway
ARNT	aryl hydrocarbon receptor nuclear translocator; HIF-1beta	Transcriptional regulator of the adaptive response to hypoxia
ARNTL	aryl hydrocarbon receptor nuclear translocator-like	Partner of CLOCK
ARRB1	Arrestin beta-1	Regulating agonist-mediated G-protein coupled receptor signaling
ASPH	aspartate beta-hydroxylase	Role in calcium homeostasis
ATG16L1	autophagy related 16-like 1, ATG16L	Autophagy
ATG4C	autophagy related 4C, cysteine peptidase	Autophagy
ATG5	autophagy related 5	Autophagy
ATXN3	ataxin 3	Deubiquitinating enzyme
AXIN1	axin1	Wnt signaling
BAG6	BCL2-associated athanogene 6	Apoptosis
BCAS1	breast carcinoma-amplified sequence 1	Candidate oncogene
BCL11B	B-cell CLL/lymphoma 11B (zinc finger protein)	P53-signaling pathway
BCL2L1	BCL2-like 1 (bcl-XL)	Apoptosis
BCL2L12	BCL2-like 12 (Proline Rich)	Apoptosis
BCL2L15	BCL2-like 15; C1orf178	Apoptosis
BCL2L2-PABPN1	read-through transcription between BCL2L2 and PABPN1 genes	Apoptosis
BCLAF1	BCL2-associated transcription factor 1	Apoptosis
BNIPL	BCL2/adenovirus E1B 19kD interacting protein like	Apoptosis
BRCA1	breast cancer 1, early onset	Tumor suppressor
BRCC3	BRCA1/BRCA2-containing complex, subunit 3	Subunit of the BRCA1-BRCA2-containing complex, E3 ubiquitin ligase
BRPF1	bromodomain and PHD finger containing, 1; Peregrin	Regulation of RUNX1 and RUNX2 transcription
BSG	basigin (Ok blood group); CD147	Stimulation of matrix metalloproteinases (MMPS) production
BTC	betacellulin	Binds EGF receptor family members
C1D	C1D Nuclear Receptor Corepressor	Apoptosis, p53 pathway
C6orf25	chromosome 6 open reading frame 25	Immunoglobulin (Ig) superfamily
CAPRIN2	caprin family member 2; C1QDC1	May be involved in apoptosis
CASC4	cancer susceptibility candidate 4	Cell proliferation and survival pathways
CASP10	caspase 10, apoptosis-related cysteine peptidase	Apoptosis
CASP7	caspase 7, apoptosis-related cysteine peptidase	Apoptosis
CASP8	caspase 8, apoptosis-related cysteine peptidase	Apoptosis
CASP9	caspase 9, apoptosis-related cysteine peptidase	Apoptosis
CAST	calpastatin	Inhibition of calpain (calcium-dependent cysteine protease)
CBFB	core-binding factor, beta subunit	Co-activator of Runx2
CD47	CD47 molecule	Integrin-associated signal transducer
CD79B	CD79b molecule, immunoglobulin-associated beta	Cytoplasmic tail containing an ITAM motif
CLEC7A	C-type lectin domain family 7, member A	TLR2-mediated activation of NF-kappa-B
CPPED1	calcineurin-like phosphoesterase domain containing 1; FLJ11151	Adipose tissue biology
CSNK1D	casein kinase 1, delta	Wnt signaling
CSNK1G3	casein kinase 1, gamma 3	Wnt signaling
DACT1	dishevelled-binding antagonist of beta-catenin 1	Wnt signaling
DAP3	death associated protein 3	Interferon-gamma-induced apoptosis
DIABLO	diablo, IAP-binding mitochondrial protein	Apoptosis
DISC1	disrupted in schizophrenia 1 protein	Modulator of the AKT-mTOR signaling
DLEC1	Deleted In Lung And Esophageal Cancer 1	Tumor supressor
DSTYK	dual serine/threonine and tyrosine protein kinase; RIPK5	Apoptosis
DUSP6	Dual Specificity Phosphatase 6	Inactivation of MAP kinases, specificity for the ERK family
DVL1	dishevelled segment polarity protein 1	Wnt signaling
ECM1	extracellular matrix protein 1	negative regulator of bone mineralization, and MMP9 proteolytic activity
ERBB2IP	erbb2 interacting protein	Ras signaling pathway
ERC1	ELKS/RAB6-interacting/CAST family member 1; RAB6IP2	Regulatory subunit of the IKK complex
EVC	Ellis van Creveld syndrome	Positive mediator of Hedgehog signaling
EXTL2	exostosin-like glycosyltransferase 2	Glycosyltransferase required for the biosynthesis of heparan-sulfate
EYA4	eyes absent homolog 4 (Drosophila)	Transcriptional activator through tyrosine phosphatase activity
FAM189B	Family With Sequence Similarity 189, Member B; C1orf2	Partner of a WW domain-containing protein involved in apoptosis
FAS	Fas cell surface death receptor; CD95	Apoptosis
FASTK	Fas-activated serine/threonine kinase; ENSG00000164896	Apoptosis
FBF1	Fas (TNFRSF6) binding factor 1	Apoptosis
FGFR1OP	Fibroblast growth factor receptor 1 oncogene partner	Proliferation
G3BP	GTPase activating protein (SH3 domain) binding protein 1	Nuclear RNA-binding protein and element of the Ras pathway
GAB1	GRB2-associated binding protein 1	EGF receptor signaling
GLYR1	glyoxylate reductase 1 homolog (Arabidopsis); N-PAC	Oxidoreductase activity, regulation of p38 MAP kinase activity
GPS1	G Protein Pathway Suppressor 1	Essential component of the COP9 signalosome complex
HGF	hepatocyte growth factor (hepapoietin A; scatter factor)	HGF
HILPDA	hypoxia inducible lipid droplet-associated; HIG2	Increases cellular lipid accumulation, and expression of IL6, MIF, VEGF
HMGXB4	HMG box domain containing 4; HMG2L1	Wnt signaling
HPS1	Hermansky-Pudlak syndrome 1	Component of multiple organelles, intracellular protein sorting
HRAS	Harvey rat sarcoma viral oncogene homolog	Ras oncogene family
IFNAR2	interferon (alpha, beta and omega) receptor 2	IFN-mediated STAT1, STAT2 and STAT3 activation
IL17RC	interleukin 17 receptor C	IL17 signaling
IL17RE	interleukin 17 receptor E	IL17 signaling
ILF3	interleukin enhancer binding factor 3, 90kDa	Subunit of NFAT
IRAK1	interleukin-1 receptor-associated kinase 1	NF-kB signaling
IRF7	interferon regulatory factor 7	Interferon signaling
JKAMP	JNK1/MAPK8-associated membrane protein; C14orf100	Regulator of MAPK8 activity
KAT6A	K(lysine) acetyltransferase 6A; MYST3	Histone acetyltransferaes, coactivator for RUNX1 and RUNX2
KIAA0101	KIAA0101	Proliferation; PCNA-Associated Factor
KITLG	Kit ligand- SCF	Ligand for the receptor-type protein-tyrosine kinase KIT
KL	Klotho	Calcium phosphore homeostasis, anti-aging hormone
L3MBTL1	L(3)Mbt-Like 1 (Drosophila)	Overexpression induces multinucleated cells
L3MBTL3	L(3)Mbt-Like 3 (Drosophila)	Polycomb group protein, repression of transcription
LAT2	linker for activation of T cells family, member 2	Adaptor between ITAM-receptor activation and downstream signaling
LDHC	lactate dehydrogenase C	Final step of anaerobic glycolysis
LGALS8	lectin, galactoside-binding, soluble, 8; galectin 8	Adhesion, apoptosis, autophagy
LGALS9	lectin, galactoside-binding, soluble, 9; galectin 9	Adhesion, apoptosis
LILRB5	leukocyte Ig-like receptor, subfamily B (with TM and ITIM domains), member 5	Immunoglobulin-like receptor (LIR) family
MADD	MAP kinase-activating death domain protein	Apoptosis
MAGED4B	Melanoma Antigen Family D, 4B	Ubiquitination
MALT1	mucosa associated lymphoid tissue lymphoma translocation gene 1	NF-kB signaling, TRAF6 partner
MAP3K3	mitogen-activated protein kinase kinase kinase 3	NF-kB and ERK signaling
MAPK7	mitogen-activated protein kinase 7	ERK signaling
MEF2C	myocyte-specific enhancer factor 2C	Survival and proliferation
MEN1	multiple endocrine neoplasia I	Transcriptional regulator, TGFb signaling, NFkB signaling
METTL13	methyltransferase like 13; KIAA0859	Apoptosis
MGRN1	mahogunin ring finger 1, E3 ubiquitin protein ligase	Ubiquitination
MIER1	mesoderm induction early response 1, transcriptional regulator	Transcription repressor
MITF	Microphthalmia-Associated Transcription Factor	Differentiating factor (inclding osteoclasts)
MLPH	melanophilin	Rab effector protein
MPZL1	myelin protein zero-like 1	Signaling induced by ConA, which includes Src family kinases
MST4	Serine/Threonine Protein Kinase MST4; MASK	Apoptosis
MTDH	metadherin	NF-kB signaling
MTUS1	microtubule associated tumor suppressor 1	Inhibition of ERK2 activation and cell proliferation
NBR1	Neighbor Of BRCA1 Gene 1	Selective autophagy
NFATC2	nuclear factor of activated T-cells, cytoplasmic, calcineurin-dependent 2	NFAT signaling
NLRP1	NLR Family, Pyrin Domain Containing 1; NALP1	Apoptosis
NLRP12	NLR Family, Pyrin Domain Containing 12; NALP12	NF-kB signaling, apoptosis
NLRP3	NLR Family, Pyrin Domain Containing 3; CIAS1	Upstream activator of NFkB signaling
OAS1	2'-5'-oligoadenylate synthetase 1, 40/46kDa	Interferon-induced antiviral enzyme, apoptosis, gene regulation
OASL	2'-5'-oligoadenylate synthetase-like	antiviral activity
OPA1	optic atrophy 1 (autosomal dominant)	Dynamin-related GTPase, mitochondrial fusion and apoptosis
OPTN	optineurin	Autophagy, membrane trafficking
OS9	Osteosarcoma Amplified 9, Endoplasmic Reticulum Lectin	Endoplasmic reticulum control, shuttle to the ubiquitination machinery
PARL	presenilin associated, rhomboid-like	Apoptosis
PDCD4	programmed cell death 4 (neoplastic transformation inhibitor)	Apoptosis
PDPK1	3-phosphoinositide dependent protein kinase-1	NF-kB and Akt signaling
PIDD	p53-induced protein with a death domain; LRDD	NF-kB signaling, apoptosis
PILRA	paired immunoglobin-like type 2 receptor alpha	cellular signaling inhibitory receptor
PILRB	paired immunoglobin-like type 2 receptor beta	cellular signaling activating receptor that associates with ITAM- adapters
PORCN	Porcupine Homolog (Drosophila)	Wnt signaling
PPARG	peroxisome proliferator-activated receptor gamma	Peroxisomal beta-oxidation pathway, NF-kB signaling
PRMT2	protein arginine methyltransferase 2	NF-kB signaling, apoptosis
PTPN13	protein tyrosine phosphatase, non-receptor type 13 (APO-1/CD95 (Fas)-associated phosphatase)	Apoptosis
PYCARD	PYD and CARD domain containing	Apoptosis
RAB3IP	Rab3A-interacting protein	activate RAB8A and RAB8B
RBCK1	RanBP-type and C3HC4-type zinc finger containing 1	Ubiquitin-protein ligase, interaction with TRAF6
RCAN1	Regulator Of Calcineurin 1; DSCR1	Inhibits calcineurin-dependent transcriptional responses
RHOT1	Ras Homolog Family Member T1	mitochondrial trafficking, apoptosis
RNF138	ring finger protein 138, E3 ubiquitin protein ligase	E3 ubiquitin-protein ligase
RREB1	Ras Responsive Element Binding Protein 1	Ras/Raf-mediated cell differentiation by enhancing calcitonin expression
RUNX2	runt-related transcription factor 2	Transcription factor involved in osteoblastic differentiation
SIRT2	Sirtuin 2- silent information regulator 2	Apoptosis, aging
SLA2	Src-like adapter protein-2	Downregulation of T / B responses, inhibition of calcium mobilization
SNAP25	synaptosomal-associated protein, 25kDa	Vesicle docking and membrane fusion
SPINT1	Serine Peptidase Inhibitor, Kunitz Type 1	Inhibitor of HGF activator
SPP1	Secreted Phosphoprotein 1	Osteopontin
STAT3	signal transducer and activator of transcription 3 (acute-phase response factor)	Signal transducer and transcription activator
SUCO	SUN Domain Containing Ossification Factor; C1orf9	Regulation of type I collagen synthesis
TBC1D25	TBC1 Domain Family, Member 25; OATL1	Autophagy
TCF4	transcription factor 4	Transcription activator
TCF7L2	transcription factor 7-like 2 (T-cell specific, HMG-box)	Wnt signaling
TCOF1	Treacher Collins-Franceschetti Syndrome 1	Nucleolar-cytoplasmic transport
TCTN3	tectonic family member 3; C10orf61	apoptosis regulation, Hedgehog signal transduction
TFEC	Transcription Factor EC	Transcriptional transactivator, interaction with MITF in target activation
THAP1	THAP Domain Containing, Apoptosis Associated Protein 1	Apoptosis
THYN1	Thymocyte nuclear protein 1	Apoptosis
TM2D2	TM2 Domain Containing 2	Apoptosis
TNFRSF10B	tumor necrosis factor receptor superfamily, member 10b; TRAIL-R2	Apoptosis
TPD52L1	Tumor protein D52-like 1	Apoptosis
TPD52L2	Tumor protein D52-like 2	Apoptosis
TRAF3	TNF receptor-associated factor 3	NF-kB signaling
TRPC4AP	transient receptor potential cation channel, subfamily C, member 4 associated protein	Ubiquitination, NF-kB signaling
TSSC4	tumor suppressing subtransferable candidate 4	tumor-suppressor
UBE2D3	Ubiquitin-Conjugating Enzyme E2D 3	Ubiquitination
UBE2V1	Ubiquitin-Conjugating Enzyme E2 Variant 1	Ubiquitination, NF-kB signaling
USP4	ubiquitin specific peptidase 4 (proto-oncogene)	Ubiquitination, NF-kB signaling
VHL	Von Hippel-Lindau Tumor Suppressor, E3 Ubiquitin Protein Ligase	Ubiquitination
WARS	tryptophanyl-tRNA synthetase	Regulation of ERK, Akt, and eNOS activation pathways
ZDHHC13	zinc finger, DHHC-type containing 13	Ion transmembrane transport, NF-kB signaling
ZDHHC16	zinc finger, DHHC-type containing 16	Apoptosis
