# Supplementary material for: Alternative splicing in osteoclasts and Paget’s disease of bone
Source: BMC Med Genet. 2014 Aug 14;15:98. doi: 10.1186/s12881-014-0098-1 (PMC4143580; doi:10.1186/s12881-014-0098-1)
Supplement: Additional file 2: Table S2. — First validation study. [file s12881-014-0098-1-S2.pdf]

Supplementary Table 2: First validation study

Student's t-test of Ψ (PSI) values for each AS event in each group (p-value) with a correction for multiple testing using the false discovery rate (q-value)
Cells with missing values are left blank
From the results of this first validation step, we selectionned 21 genes with significant p-value<0.05 (highlighted in yellow) in at least one comparison and acceptable q-value (less that 55% false positive)

PBD^{wt}	PDB no mutation	(n=5)
PBD^{P392L}	PDB with SQSTM1/p62 P392L mutation	(n=5)
HD^{wt}	HD no mutation	(n=5)
HD^{P392L}	HD with SQSTM1/p62 P392L mutation	(n=5)

Gene	Primer Pair	Paget vs No Paget p-value	Paget vs No Paget q-value	Mutation vs No Mutation p-value	Mutation vs No Mutation q-value	PDB^{wt} vs PDB^{P392L} p-value	PDB^{wt} vs PDB^{P392L} q-value	PDB^{wt} vs HD^{wt} p-value	PDB^{wt} vs HD^{wt} q-value	PBD^{wt} vs HD^{P392L} p-value	PBD^{wt} vs HD^{P392L} q-value	PDB^{P392L} vs HD^{wt} p-value	PDB^{P392L} vs HD^{wt} q-value	PDB^{P392L} vs HD^{P392L} p-value	PDB^{P392L} vs HD^{P392L} q-value	HD^{wt} vs HD^{P392L} p-value	HD^{wt} vs HD^{P392L} q-value
ABI1	rs.ABI1.F1 rs.ABI1.R1	2,364E-1	4,786E-1	7,504E-1	9,923E-1	2,275E-1	8,969E-1	1,808E-2	1,774E-1	2,981E-1	6,435E-1	5,548E-1	8,401E-1	8,708E-1	8,853E-1	4,349E-1	7,584E-1
ABTB1	refseq_ABTB1.F1 refseq_ABTB1.R1	5,035E-1	4,861E-1	4,636E-1	9,923E-1	2,797E-1	8,969E-1	8,212E-2	4,028E-1	9,650E-1	7,156E-1	7,176E-2	7,446E-1	3,386E-2	5,220E-1	2,614E-3	2,214E-1
AKT2	AKT2.e02.F1 AKT2.e02.R1	7,419E-1	9,953E-1	7,588E-1	9,788E-1	1,745E-1	5,964E-1	3,570E-1	7,899E-1	5,988E-1	9,620E-1	9,881E-1	9,916E-1	1,435E-1	7,950E-1	2,471E-1	9,005E-1
ANXA6	refseq_ANXA6.F1 refseq_ANXA6.R1	3,750E-1	4,786E-1	4,480E-1	9,923E-1	7,784E-1	9,757E-1	6,699E-1	6,479E-1	2,718E-1	6,435E-1	9,355E-1	9,130E-1	4,693E-1	8,743E-1	4,783E-1	7,981E-1
APAF1	APAF1.F8 APAF1.R8	5,599E-1	9,953E-1	6,795E-1	9,788E-1	2,634E-1	5,964E-1	2,201E-1	7,899E-1	6,109E-1	9,620E-1	7,606E-1	9,916E-1	6,069E-1	8,089E-1	5,238E-1	9,312E-1
APAF1	rs.APAF1.F1 rs.APAF1.R1	7,554E-1	9,953E-1	7,174E-1	9,788E-1	2,787E-1	5,964E-1	2,922E-1	7,899E-1	7,180E-1	9,620E-1	9,489E-1	9,916E-1	4,050E-1	7,950E-1	4,275E-1	9,205E-1
APC	APC.F16 APC.R14	5,180E-1	9,953E-1	8,702E-1	9,788E-1	7,760E-1	7,073E-1	3,963E-1	7,899E-1	7,870E-1	9,620E-1	4,938E-1	9,916E-1	9,923E-1	8,929E-1	5,082E-1	9,205E-1
ARNT	refseq_ARNT.F1 refseq_ARNT.R1	8,722E-1	9,953E-1	7,128E-1	9,788E-1	5,133E-1	6,145E-1	4,117E-1	7,899E-1	7,224E-1	9,620E-1	8,814E-1	9,916E-1	2,844E-1	7,950E-1	2,011E-1	9,005E-1
ARNTL	refseq_ARNTL.F1 refseq_ARNTL.R1	6,700E-1	9,953E-1	9,413E-1	9,788E-1	5,456E-1	6,216E-1	7,470E-1	9,691E-1	8,474E-1	9,620E-1	6,300E-1	9,916E-1	2,432E-1	7,950E-1	4,476E-1	9,205E-1
ARRB1	refseq_ARRB1.F1 refseq_ARRB1.R1	9,044E-1	9,953E-1	5,567E-1	9,788E-1	7,565E-1	7,073E-1	9,918E-1	9,942E-1	6,524E-1	9,620E-1	7,524E-1	9,916E-1	8,945E-1	8,789E-1	6,443E-1	9,757E-1
ASPH	refseq_ASPH.F1 refseq_ASPH.R1	3,763E-1	4,786E-1	8,294E-1	9,923E-1	9,689E-1	9,764E-1	1,398E-1	4,672E-1	6,459E-1	6,435E-1	6,770E-1	8,768E-1	8,287E-1	8,852E-1	6,188E-1	8,798E-1
ATG16L1	refseq_ATG16L1.F1 refseq_ATG16L1.R1	6,201E-1	9,953E-1	2,262E-1	9,788E-1	4,786E-1	6,145E-1	7,086E-1	9,617E-1	3,958E-1	9,620E-1	3,971E-1	9,916E-1	7,568E-1	8,448E-1	2,300E-1	9,005E-1
ATG4C	refseq_ATG4C.F1 refseq_ATG4C.R1	6,970E-1	9,953E-1	5,897E-2	9,788E-1	1,195E-1	5,964E-1	3,659E-1	7,899E-1	1,406E-1	9,620E-1	3,131E-1	9,916E-1	9,173E-1	8,849E-1	3,658E-1	9,005E-1
ATG5	APG5L.u.f.9 APG5L.u.r.5	9,565E-1	9,953E-1	9,686E-1	9,788E-1	8,054E-1	7,162E-1	7,634E-1	9,771E-1	9,937E-1	9,986E-1	9,209E-1	9,916E-1	7,069E-1	8,351E-1	6,349E-1	9,757E-1
ATG5	ATG5.e06.F1 ATG5.e06.R1	5,319E-2	9,953E-1	4,328E-1	9,788E-1	3,958E-1	6,145E-1	1,640E-2	5,608E-1	2,721E-1	9,620E-1	8,310E-2	9,380E-1	9,724E-1	8,929E-1	4,430E-2	8,506E-1
ATXN3	refseq_ATXN3.F1 refseq_ATXN3.R1	2,468E-1	9,953E-1	3,440E-1	9,788E-1	9,550E-2	5,964E-1	1,451E-1	7,128E-1	2,104E-1	9,620E-1	8,619E-1	9,916E-1	9,902E-1	8,929E-1	9,091E-1	9,757E-1
ATXN3	rs.ATXN3.F1 rs.ATXN3.R1	4,586E-1	9,953E-1	5,618E-1	9,788E-1	3,871E-1	6,145E-1	4,531E-1	7,899E-1	3,741E-1	9,620E-1	8,960E-1	9,916E-1	4,371E-2	7,505E-1	6,244E-2	8,888E-1
AXIN1	AXIN1.u.f.11 AXIN1.R5	3,079E-1	9,953E-1	8,277E-1	9,788E-1	2,267E-1	5,964E-1	6,239E-2	5,608E-1	6,642E-1	9,620E-1	3,934E-2	8,278E-1	5,673E-1	8,089E-1	1,948E-1	9,005E-1
AXIN1	rs.AXIN1.F1 rs.AXIN1.R1	9,940E-1	9,953E-1	7,039E-1	9,788E-1	1,730E-1	5,964E-1	2,320E-1	7,899E-1	8,318E-1	9,620E-1	4,904E-1	9,916E-1	1,377E-1	7,950E-1	2,019E-1	9,005E-1
BAG6	refseq_BAT3.F1 refseq_BAT3.R1	5,714E-1	9,953E-1	2,058E-1	9,788E-1	2,074E-1	5,964E-1	8,685E-1	9,809E-1	6,391E-1	9,620E-1	2,236E-1	9,916E-1	2,255E-1	7,950E-1	7,524E-1	9,757E-1
BCAS1	BCAS1.F9 BCAS1.R4	3,996E-1	4,786E-1	7,753E-2	9,923E-1					2,088E-1	6,435E-1						
BCL11B	refseq_BCL11B.F2 refseq_BCL11B.R2	6,283E-1	4,972E-1	2,442E-1	9,923E-1	7,463E-1	9,757E-1	1,084E-1	4,654E-1	6,136E-1	6,435E-1	2,162E-1	7,542E-1	5,186E-1	8,743E-1	1,754E-1	7,559E-1
BCL2L1	bcl2l1.f.2 bcl2l1.r.2	9,268E-1	9,953E-1	7,274E-2	9,788E-1	2,517E-1	5,964E-1	8,646E-1	9,809E-1	3,476E-1	9,620E-1	1,114E-1	9,380E-1	5,079E-1	8,075E-1	1,725E-1	9,005E-1
BCL2L12	BCL2L12-3 BCL2L12-4	3,636E-1	4,786E-1	7,226E-1	9,923E-1	1,352E-1	7,253E-1	3,627E-1	5,877E-1	2,875E-1	6,435E-1	6,872E-1	8,768E-1	9,990E-3	4,874E-1	7,884E-2	6,709E-1
BCL2L15*	refseq_C1orf178.F1 refseq_C1orf178.R1	9,128E-3	3,493E-1	4,193E-1	9,923E-1	7,921E-1	9,757E-1	5,224E-2	2,990E-1	3,561E-1	6,435E-1	4,448E-3	3,838E-1	2,875E-1	8,366E-1	6,562E-1	8,798E-1
BCL2L2andPABPN	BCL2L2andPABPN1.e02.F1 BCL2L2andF	3,613E-1	4,786E-1	7,894E-1	9,923E-1	9,709E-1	9,764E-1	3,120E-1	5,790E-1	5,054E-1	6,435E-1	5,405E-1	8,401E-1	7,469E-1	8,743E-1	6,436E-1	8,798E-1
BCLAF1	rs.BCLAF1.F1 rs.BCLAF1.R1	9,504E-1	9,953E-1	6,965E-1	9,788E-1	4,540E-1	6,145E-1	3,187E-1	7,899E-1	8,680E-1	9,689E-1	1,256E-1	9,380E-1	2,759E-1	7,950E-1	1,775E-1	9,005E-1
BNIPL	refseq_BNIPL.F1 refseq_BNIPL.R1	6,196E-1	4,972E-1	1,031E-1	9,923E-1	8,929E-1	9,764E-1	1,757E-1	4,805E-1	3,739E-1	6,435E-1	3,220E-1	7,651E-1	5,000E-1	8,743E-1	5,877E-2	6,709E-1
BRCA1	rs.BRCA1.F1 rs.BRCA1.R1	7,335E-1	4,972E-1	8,875E-1	9,923E-1	5,422E-1	9,107E-1	3,415E-1	5,827E-1	9,161E-1	6,989E-1	5,822E-1	8,485E-1	5,924E-1	8,743E-1	3,563E-1	7,559E-1
BRCC3	refseq_BRCC3.F1 refseq_BRCC3.R1	6,242E-1	9,953E-1	2,536E-1	9,788E-1	1,165E-1	5,964E-1	4,187E-1	7,899E-1	7,044E-1	9,620E-1	7,953E-2	9,380E-1	1,871E-2	7,505E-1	3,386E-1	9,005E-1
BRPF1	refseq_BRPF1.F2 refseq_BRPF1.R2	4,537E-1	9,953E-1	7,845E-1	9,788E-1	2,156E-1	5,964E-1	1,415E-1	7,128E-1	5,817E-1	9,620E-1	4,431E-1	9,916E-1	4,587E-1	7,950E-1	2,902E-1	9,005E-1
BSG	refseq_BSG.F3 refseq_BSG.R3	3,453E-1	9,953E-1	4,200E-2	9,788E-1	6,232E-1	6,229E-1	2,287E-2	5,608E-1	2,386E-1	9,620E-1	3,292E-2	8,278E-1	4,223E-2	7,505E-1	7,415E-3	3,559E-1
BTC	BTC.F1 BTC.R1	7,322E-1	4,972E-1	1,564E-2	7,349E-1	1,806E-2	4,077E-1	4,900E-1	6,224E-1	5,653E-3	1,941E-1	1,752E-1	7,446E-1	5,798E-1	8,743E-1	1,143E-1	7,559E-1
C1D	rs.C1D.F1 rs.C1D.R1	5,588E-1	4,957E-1	2,767E-1	9,923E-1	6,994E-1	9,757E-1	4,606E-1	6,224E-1	1,105E-1	6,435E-1	4,269E-1	7,651E-1	1,366E-1	6,781E-1	3,389E-1	7,559E-1
C6orf25	rs.C6orf25.F1 rs.C6orf25.R1	5,975E-1	4,972E-1	6,611E-1	9,923E-1	5,292E-1	9,107E-1	5,251E-1	6,315E-1	6,575E-1	6,435E-1	9,522E-1	9,130E-1	9,644E-1	8,853E-1	9,462E-1	8,955E-1
CAPRIN2	refseq_C1QDC1.F1 refseq_C1QDC1.R1	7,388E-1	4,972E-1	4,475E-1	9,923E-1	9,301E-1	9,764E-1	8,116E-1	7,037E-1	4,980E-1	6,435E-1	7,629E-1	8,860E-1	5,495E-1	8,743E-1	4,276E-1	7,584E-1
CAPRIN2	refseq_C1QDC1.F3 refseq_C1QDC1.R3	9,504E-1	5,523E-1	3,111E-1	9,923E-1	9,270E-2	6,675E-1	2,188E-1	4,805E-1	4,957E-1	6,435E-1	4,164E-1	7,651E-1	1,124E-1	6,730E-1	3,004E-1	7,559E-1
CASC4	CASC4.F1 NM_177974-R1	1,718E-1	4,786E-1	5,805E-1	9,923E-1	2,659E-2	4,077E-1	6,528E-4	2,241E-2	1,878E-1	6,435E-1	4,521E-1	7,651E-1	5,097E-1	8,743E-1	2,052E-1	7,559E-1
CASC4	rs.CASC4.F1 rs.CASC4.R1	2,663E-1	4,786E-1	4,070E-1	9,923E-1	1,181E-1	7,253E-1	1,534E-2	1,756E-1	3,519E-1	6,435E-1	6,838E-1	8,768E-1	6,902E-1	8,743E-1	7,879E-1	8,932E-1
CASP10	rs.CASP10.F1 rs.CASP10.R1	4,043E-1	9,953E-1	5,912E-1	9,788E-1	4,274E-1	6,145E-1	3,343E-1	7,899E-1	5,061E-1	9,620E-1	5,125E-1	9,916E-1	9,508E-1	8,929E-1	8,907E-1	9,757E-1
CASP7	rs.CASP7.F1 rs.CASP7.R1	3,021E-1	4,786E-1	7,898E-1	9,923E-1	4,137E-1	9,107E-1	4,985E-1	6,224E-1	6,010E-2	6,435E-1	7,096E-1	8,860E-1	4,158E-1	8,743E-1	9,731E-1	8,955E-1
CASP8	rs.CASP8.F2 rs.CASP8.R2	2,742E-1	9,953E-1	7,524E-1	9,788E-1	4,834E-1	6,145E-1	8,305E-1	9,809E-1	3,196E-1	9,620E-1	5,982E-1	9,916E-1	6,538E-2	7,950E-1	1,842E-1	9,005E-1
CASP9	primer-113212 primer-113213	7,923E-1	9,953E-1	9,356E-1	9,788E-1	8,551E-1	7,195E-1	7,862E-1	9,809E-1	8,055E-1	9,620E-1	9,128E-1	9,916E-1	9,778E-1	8,929E-1	9,111E-1	9,757E-1
CASP9	rs.CASP9.F1 rs.CASP9.R1	8,591E-1	9,953E-1	8,417E-1	9,788E-1	8,905E-1	7,304E-1	7,210E-1	9,617E-1	9,879E-1	9,986E-1	8,107E-1	9,916E-1	8,740E-1	8,789E-1	7,027E-1	9,757E-1
CAST	rs.CAST.F1 rs.CAST.R1	7,471E-1	9,953E-1	7,611E-1	9,788E-1	9,368E-1	7,304E-1	9,316E-1	9,884E-1	6,126E-1	9,620E-1	9,916E-1	9,916E-1	6,484E-1	8,166E-1	5,685E-1	9,747E-1
CAST	rs.CAST.F2 rs.CAST.R2	7,724E-1	9,953E-1	4,525E-1	9,788E-1	7,929E-1	7,138E-1	9,669E-1	9,884E-1	4,891E-1	9,620E-1	7,642E-1	9,916E-1	7,101E-1	8,351E-1	4,624E-1	9,205E-1
CBFB	refseq_CBFB.F1 refseq_CBFB.R1	7,916E-1	9,953E-1	2,862E-1	9,788E-1	8,585E-1	7,195E-1	3,638E-1	7,899E-1	6,778E-1	9,620E-1	5,713E-2	9,141E-1	3,421E-1	7,950E-1	7,470E-2	8,888E-1
CD47	rs.CD47.F1 rs.CD47.R1	2,662E-1	9,953E-1	9,167E-1	9,788E-1	6,236E-1	6,229E-1	7,420E-1	9,691E-1	4,669E-1	9,620E-1	4,520E-1	9,916E-1	3,132E-1	7,950E-1	6,015E-1	9,757E-1
CD79B	refseq_CD79B.F1 refseq_CD79B.R1	1,999E-1	9,953E-1	3,965E-1	9,788E-1	9,417E-1	7,304E-1	1,654E-1	7,217E-1	7,993E-1	9,620E-1	1,025E-1	9,380E-1	7,281E-1	8,447E-1	2,502E-1	9,005E-1
CLEC7A	refseq_CLEC7A.F1 refseq_CLEC7A.R1	1,313E-1	4,786E-1	5,094E-1	9,923E-1	2,567E-1	8,969E-1	4,445E-1	6,224E-1	4,589E-1	6,435E-1	2,454E-1	7,651E-1	1,582E-1	6,830E-1	7,750E-1	8,932E-1
CPPED1	rs.FLJ11151.F1 rs.FLJ11151.R1	5,541E-1	9,953E-1	3,322E-1	9,788E-1	1,025E-1	5,964E-1	4,419E-1	7,899E-1	8,363E-1	9,620E-1	3,982E-2	8,278E-1	1,367E-1	7,950E-1	5,927E-1	9,757E-1
CSNK1D	refseq_CSNK1D.F2 refseq_CSNK1D.R2	8,874E-1	9,953E-1	5,043E-1	9,788E-1	2,070E-1	5,964E-1	3,105E-1	7,899E-1	6,705E-1	9,620E-1	4,081E-1	9,916E-1	2,986E-1	7,950E-1	4,846E-1	9,205E-1
CSNK1G3	refseq_CSNK1G3.F3 refseq_CSNK1G3.R	3,972E-1	9,953E-1	5,728E-1	9,788E-1	3,418E-1	6,145E-1	2,045E-1	7,851E-1	4,147E-1	9,620E-1	8,256E-1	9,916E-1	9,009E-1	8,789E-1	9,737E-1	9,890E-1
DACT1	rs.DACT1.F1 rs.DACT1.R1	3,187E-1	9,953E-1	9,292E-1	9,788E-1	5,208E-1	6,145E-1	9,679E-1	9,884E-1	4,729E-1	9,620E-1	5,223E-1	9,916E-1	1,601E-1	7,950E-1	5,488E-1	9,579E-1
DAP3	refseq_DAP3.F2 refseq_DAP3.R2	1,918E-1	4,786E-1	6,608E-1	9,923E-1	5,648E-1	9,107E-1	8,266E-1	7,037E-1	3,773E-1	6,435E-1	1,897E-1	7,446E-1	2,171E-1	8,171E-1	3,996E-1	7,559E-1
DIABLO	rs.DIABLO.F1 rs.DIABLO.R1	9,155E-1	9,953E-1	7,237E-1	9,788E-1	4,565E-1	6,145E-1	5,618E-1	8,626E-1	8,065E-1	9,620E-1	7,989E-1	9,916E-1	5,837E-1	8,089E-1	7,234E-1	9,757E-1
DISC1	refseq_DISC1.F2 refseq_DISC1.R2	7,473E-1	9,953E-1	6,729E-1	9,788E-1	6,848E-1	6,748E-1	4,949E-1	8,192E-1	6,082E-1	9,620E-1	9,468E-1	9,916E-1	4,530E-1	7,950E-1	3,219E-1	9,005E-1
DLEC1	refseq_DLEC1.F1 refseq_DLEC1.R1	4,184E-1	4,786E-1	4,459E-1	9,923E-1	9,764E-1	9,764E-1	9,582E-1	7,356E-1	2,852E-1	6,435E-1	9,755E-1	9,151E-1	2,421E-1	8,171E-1	3,598E-1	7,559E-1
DUSP6	rs.DUSP6.F1 rs.DUSP6.R1	1,270E-1	4,786E-1	9,940E-1	9,940E-1	3,791E-1	9,107E-1	1,344E-1	4,672E-1	2,842E-1	6,435E-1	3,196E-1	7,651E-1	7,832E-1	8,743E-1	4,018E-1	7,559E-1
DVL1	DVL1.e02.F1 DVL1.e02.R1	6,372E-1	9,953E-1	2,977E-1	9,788E-1	5,193E-1	6,145E-1	8,429E-1	9,809E-1	4,061E-1	9,620E-1	6,237E-1	9,916E-1	6,482E-1	8,166E-1	4,754E-1	9,205E-1
ECM1	refseq_ECM1.F2 refseq_ECM1.R2	3,541E-1	4,786E-1	5,637E-1	9,923E-1	7,241E-1	9,757E-1	5,366E-1	6,315E-1	7,824E-1	6,587E-1	3,964E-1	7,651E-1	5,159E-1	8,743E-1	6,683E-1	8,798E-1
ERBB2IP	refseq_ERBB2IP.F1 refseq_ERBB2IP.R1	5,679E-1	4,957E-1	8,081E-1	9,923E-1	4,217E-1	9,107E-1	9,501E-1	7,356E-1	8,682E-1	6,986E-1	3,561E-1	7,651E-1	5,867E-1	8,743E-1	8,878E-1	8,932E-1
ERC1	refseq_RAB6IP2.F5 refseq_RAB6IP2.R5	9,455E-1	5,523E-1	8,382E-1	9,923E-1	6,039E-1	9,107E-1	7,775E-1	7,037E-1	9,149E-1	6,989E-1	8,744E-1	9,130E-1	6,659E-1	8,743E-1	8,394E-1	8,932E-1
EVC	EVC.e05.F1 EVC.e05.R1	2,923E-1	4,786E-1	9,327E-1	9,923E-1	9,433E-1	9,764E-1	4,903E-1	6,224E-1	5,576E-1	6,435E-1	3,251E-1	7,651E-1	4,736E-1	8,743E-1	9,539E-1	8,955E-1
EXTL2	refseq_EXTL2.F2 refseq_EXTL2.R2	6,480E-1	4,972E-1	3,157E-1	9,923E-1	1,354E-1	7,253E-1	1,565E-1	4,672E-1	3,252E-1	6,435E-1	6,909E-1	8,768E-1	5,091E-1	8,743E-1	7,028E-1	8,798E-1
EYA4	refseq_EYA4.F1 refseq_EYA4.R1	7,322E-1	4,972E-1	2,999E-1	9,923E-1	7,527E-1	9,757E-1	8,720E-1	7,129E-1	3,150E-1	6,435E-1	6,722E-1	8,768E-1	5,507E-1	8,743E-1	3,047E-1	7,559E-1
FAM189B	refseq_C1orf2.F1 refseq_C1orf2.R1	3,131E-1	4,786E-1	1,003E-1	9,923E-1	8,517E-2	6,675E-1	5,692E-1	6,315E-1	4,520E-1	6,435E-1	1,880E-1	7,446E-1	1,276E-1	6,730E-1	3,669E-1	7,559E-1
FAS	rs.FAS.F2 rs.FAS.R2	4,035E-1	9,953E-1	6,116E-1	9,788E-1	8,023E-2	5,964E-1	5,805E-2	5,608E-1	4,551E-1	9,620E-1	4,922E-1	9,916E-1	3,993E-1	7,950E-1	2,936E-1	9,005E-1
FASTK	FAST_HUMAN-5 FAST_HUMAN-6	3,640E-1	4,786E-1	9,136E-1	9,923E-1	6,524E-1	9,680E-1	3,446E-1	5,827E-1	6,352E-1	6,435E-1	4,379E-1	7,651E-1	9,401E-1	8,853E-1	5,109E-1	8,235E-1
FBF1	refseq_FBF1.F1 refseq_FBF1.R1	4,997E-1	4,861E-1	3,608E-1	9,923E-1	4,095E-1	9,107E-1	5,789E-1	6,315E-1	2,762E-1	6,435E-1	8,797E-1	9,130E-1	7,471E-1	8,743E-1	7,066E-1	8,798E-1
FGFR1OP	FGFR1OP.u.f.10 FGFR1OP.u.r.12	9,636E-1	5,523E-1	5,293E-1	9,923E-1	7,954E-1	9,757E-1	8,409E-1	7,042E-1	7,506E-1	6,587E-1	5,510E-1	8,401E-1	8,897E-1	8,853E-1	5,878E-1	8,798E-1
G3BP	refseq_G3BP.F2 refseq_G3BP.R2	1,534E-1	4,786E-1	8,657E-1	9,923E-1	5,685E-1	9,107E-1	7,169E-1	6,744E-1	5,191E-1	6,435E-1	5,536E-2	7,446E-1	1,075E-1	6,730E-1	5,864E-1	8,798E-1
GAB1	refseq_GAB1.F2 refseq_GAB1.R2	6,023E-1	4,972E-1	2,417E-1	9,923E-1	1,991E-2	4,077E-1	3,705E-1	5,877E-1	6,800E-1	6,435E-1	1,818E-1	7,446E-1	2,399E-1	8,171E-1	8,405E-1	8,932E-1
GLYR1	N-PAC.e04.F1 N-PAC.e04.R1	4,619E-1	9,953E-1	8,970E-1	9,788E-1	2,713E-1	5,964E-1	1,311E-1	7,128E-1	7,385E-1	9,620E-1	1,270E-1	9,380E-1	2,763E-1	7,950E-1	9,083E-2	8,888E-1
GPS1	rs.GPS1.F1 rs.GPS1.R1	7,767E-1	9,953E-1	5,473E-1	9,788E-1	4,237E-1	6,145E-1	5,146E-1	8,373E-1	6,523E-1	9,620E-1	6,879E-1	9,916E-1	7,061E-1	8,351E-1	8,729E-1	9,757E-1
HGF	rs.HGF.F1 rs.HGF.R1	6,978E-1	9,953E-1	6,441E-1	9,788E-1	5,937E-1	6,229E-1	9,491E-1	9,884E-1	9,633E-1	9,986E-1	5,560E-1	9,916E-1	6,614E-1	8,166E-1	9,174E-1	9,757E-1
HILPDA	rs.HIG2.F1 rs.HIG2.R1	3,142E-1	9,953E-1	1,188E-1	9,788E-1	1,994E-1	5,964E-1	3,937E-1	7,899E-1	1,241E-1	9,620E-1	6,767E-1	9,916E-1	5,889E-1	8,089E-1	4,091E-1	9,205E-1
HMGXB4	refseq_HMG2L1.F1 refseq_HMG2L1.R1	9,559E-1	9,953E-1	1,716E-1	9,788E-1	1,107E-1	5,964E-1	3,063E-1	7,899E-1	4,522E-1	9,620E-1	2,265E-2	8,278E-1	1,617E-1	7,950E-1	7,318E-1	9,757E-1
HMGXB4	refseq_HMG2L1.F3 refseq_HMG2L1.R3	4,298E-1	9,953E-1	3,620E-1	9,788E-1	5,225E-1	6,145E-1	5,585E-1	8,626E-1	4,331E-1	9,620E-1	4,620E-1	9,916E-1	2,083E-1	7,950E-1	1,026E-1	8,956E-1
HPS1	refseq_HPS1.F2 refseq_HPS1.R2	1,688E-1	4,786E-1	6,404E-1	9,923E-1	8,296E-1	9,764E-1	3,479E-1	5,827E-1	3,230E-1	6,435E-1	4,005E-1	7,651E-1	3,685E-1	8,743E-1	6,879E-1	8,798E-1
HPS1	refseq_HPS1.F4 refseq_HPS1.R4	7,058E-1	4,972E-1	4,002E-1	9,923E-1	6,877E-1	9,757E-1	1,996E-1	4,805E-1	7,728E-1	6,587E-1	3,316E-1	7,651E-1	4,535E-1	8,743E-1	7,275E-2	6,709E-1
HRAS	rs.HRAS.F1 rs.HRAS.R1	5,678E-1	4,957E-1	9,149E-1	9,923E-1	2,544E-1	8,969E-1	1,513E-1	4,672E-1	7,866E-1	6,587E-1	4,498E-1	7,651E-1	2,740E-1	8,366E-1	1,503E-1	7,559E-1
IFNAR2	refseq_IFNAR2.F1 refseq_IFNAR2.R1	6,228E-1	9,953E-1	4,920E-1	9,788E-1	7,599E-1	7,073E-1	3,225E-1	7,899E-1	9,103E-1	9,867E-1	3,007E-1	9,916E-1	6,074E-1	8,089E-1	2,034E-1	9,005E-1
IL17RC	refseq_IL17RC.F2 refseq_IL17RC.R2	3,488E-1	9,953E-1	3,635E-1	9,788E-1	2,734E-1	5,964E-1	2,593E-1	7,899E-1	3,055E-1	9,620E-1	9,773E-1	9,916E-1	7,927E-1	8,498E-1	7,397E-1	9,757E-1
IL17RE	rs.IL17RE.F1 rs.IL17RE.R1	9,163E-1	9,953E-1	6,282E-2	9,788E-1	2,399E-1	5,964E-1	9,674E-1	9,884E-1	2,213E-1	9,620E-1	2,304E-1	9,916E-1	7,799E-1	8,498E-1	2,122E-1	9,005E-1
ILF3	rs.ILF3.F1 rs.ILF3.R1	5,802E-1	9,953E-1	7,758E-2	9,788E-1	4,394E-1	6,145E-1	4,033E-1	7,899E-1	3,773E-1	9,620E-1	1,118E-1	9,380E-1	9,333E-1	8,903E-1	9,258E-2	8,888E-1
IRAK1	rs.IRAK1.F2 rs.IRAK1.R2	9,315E-1	9,953E-1	1,578E-1	9,788E-1	1,370E-1	5,964E-1	3,491E-1	7,899E-1	3,243E-1	9,620E-1	3,419E-1	9,916E-1	3,565E-1	7,950E-1	9,521E-1	9,828E-1
IRF7	refseq_IRF7.F1 refseq_IRF7.R1	3,880E-1	4,786E-1	6,152E-2	9,923E-1	9,602E-1	9,764E-1	2,855E-1	5,446E-1	1,293E-1	6,435E-1	1,410E-1	7,446E-1	1,265E-1	6,730E-1	5,698E-2	6,709E-1
JKAMP	rs.C14orf100.F1 rs.C14orf100.R1	4,094E-1	9,953E-1	6,017E-1	9,788E-1	4,094E-1	6,145E-1	8,650E-2	5,931E-1	8,296E-1	9,620E-1	3,144E-1	9,916E-1	4,346E-1	7,950E-1	4,420E-2	8,506E-1
KAT6A	rs.MYST3.F1 rs.MYST3.R1	7,286E-2	9,953E-1	6,596E-1	9,788E-1	2,863E-1	5,964E-1	5,986E-1	8,840E-1	1,651E-1	9,620E-1	2,429E-1	9,916E-1	3,128E-2	7,505E-1	8,181E-1	9,757E-1
KIAA0101	refseq_KIAA0101.F1 refseq_KIAA0101.R	7,673E-1	5,041E-1	3,905E-1	9,923E-1	9,132E-1	9,764E-1	5,886E-1	6,315E-1	7,863E-1	6,587E-1	1,614E-1	7,446E-1	7,539E-1	8,743E-1	2,396E-1	7,559E-1
KITLG	KITLG.u.f.9 KITLG.u.r.10	4,225E-1	9,953E-1	9,055E-1	9,788E-1	9,951E-1	7,590E-1	7,039E-1	9,617E-1	4,662E-1	9,620E-1	6,981E-1	9,916E-1	4,525E-1	7,950E-1	8,953E-1	9,757E-1
KITLG	rs.KITLG.F1 rs.KITLG.R1	2,221E-1	9,953E-1	5,325E-1	9,788E-1	8,983E-1	7,304E-1	1,559E-1	7,128E-1	6,209E-1	9,620E-1	2,862E-1	9,916E-1	8,039E-1	8,498E-1	2,714E-1	9,005E-1
KL	refseq_KL.F2 refseq_KL.R2	4,159E-1	9,953E-1	5,085E-1	9,788E-1	3,073E-2	5,964E-1	2,609E-2	5,608E-1	3,661E-1	9,620E-1	8,482E-1	9,916E-1	3,306E-1	7,950E-1	2,910E-1	9,005E-1
L3MBTL1	refseq_L3MBTL.F1 refseq_L3MBTL.R1	4,699E-1	4,861E-1	3,873E-1	9,923E-1	9,432E-2	6,675E-1	6,470E-1	6,464E-1	9,063E-1	6,989E-1	3,277E-1	7,651E-1	3,711E-2	5,220E-1	6,655E-1	8,798E-1
L3MBTL1	refseq_L3MBTL.F1 refseq_L3MBTL.R1	6,550E-1	9,953E-1	4,588E-2	9,788E-1	7,744E-3	5,647E-1	5,678E-2	5,608E-1	2,496E-2	9,620E-1	2,927E-1	9,916E-1	1,738E-1	7,950E-1	8,747E-1	9,757E-1
L3MBTL3	refseq_L3MBTL3.F2 refseq_L3MBTL3.R2	9,839E-1	9,953E-1	8,778E-1	9,788E-1	1,798E-1	5,964E-1	9,141E-3	5,608E-1	7,940E-1	9,620E-1	9,337E-1	9,916E-1	1,511E-1	7,950E-1	2,983E-3	2,863E-1
LAT2	refseq_LAT2.F1 refseq_LAT2.R1	8,669E-1	9,953E-1	8,883E-1	9,788E-1	2,259E-1	5,964E-1	2,021E-1	7,851E-1	8,295E-1	9,620E-1	9,834E-1	9,916E-1	1,949E-1	7,950E-1	1,542E-1	9,005E-1
LDHC	LDHC.e01.F1 LDHC.e01.R1	4,069E-1	4,786E-1	9,460E-1	9,923E-1					7,761E-1	6,587E-1						
LGALS8	refseq_LGALS8.F2 refseq_LGALS8.R2	1,896E-2	3,493E-1	1,121E-1	9,923E-1	2,246E-2	4,077E-1	4,465E-1	6,224E-1	4,201E-1	6,435E-1	1,950E-2	5,166E-1	1,155E-2	4,874E-1	8,655E-1	8,932E-1
LGALS9	LGALS9.F10 LGALS9.R6	3,938E-1	4,786E-1	1,882E-1	9,923E-1	1,438E-1	7,253E-1	4,794E-2	2,990E-1	1,598E-1	6,435E-1	7,514E-1	8,860E-1	9,488E-1	8,853E-1	8,119E-1	8,932E-1
LGALS9	rs.LGALS9.F1 rs.LGALS9.R1	4,981E-1	4,861E-1	2,688E-1	9,923E-1	2,200E-1	8,969E-1	8,076E-2	4,028E-1	2,123E-1	6,435E-1	7,853E-1	8,860E-1	8,969E-1	8,853E-1	8,912E-1	8,932E-1
LILRB5	rs.LILRB5.F1 rs.LILRB5.R1	3,239E-2	9,953E-1	5,474E-1	9,788E-1	4,588E-1	6,145E-1	1,392E-1	7,128E-1	1,631E-1	9,620E-1	4,312E-2	8,278E-1	1,999E-1	7,950E-1	9,787E-1	9,890E-1
MADD	refseq_MADD.F2 refseq_MADD.R2	8,817E-1	9,953E-1	4,925E-1	9,788E-1	1,862E-1	5,964E-1	2,958E-1	7,899E-1	6,548E-1	9,620E-1	4,175E-1	9,916E-1	2,254E-1	7,950E-1	4,315E-1	9,205E-1
MADD	refseq_MADD.F3 refseq_MADD.R3	7,377E-1	9,953E-1	9,856E-1	9,856E-1	4,073E-1	6,145E-1	3,366E-1	7,899E-1	8,462E-1	9,620E-1	7,403E-1	9,916E-1	4,512E-1	7,950E-1	3,658E-1	9,005E-1
MAGED4B	rs.MAGED4B.F1 rs.MAGED4B.R1	9,804E-2	4,786E-1	7,605E-1	9,923E-1	8,148E-1	9,764E-1	1,484E-1	4,672E-1	4,419E-1	6,435E-1	1,266E-1	7,446E-1	5,279E-1	8,743E-1	3,570E-1	7,559E-1
MALT1	refseq_MALT1.F1 refseq_MALT1.R1	1,687E-1	4,786E-1	3,593E-1	9,923E-1	7,498E-1	9,757E-1	9,401E-2	4,304E-1	6,484E-1	6,435E-1	1,895E-1	7,446E-1	9,652E-1	8,853E-1	1,661E-1	7,559E-1
MAP3K3	refseq_MAP3K3.F2 refseq_MAP3K3.R2	2,393E-2	9,953E-1	3,104E-1	9,788E-1	2,429E-1	5,964E-1	4,345E-2	5,608E-1	4,909E-2	9,620E-1	3,169E-1	9,916E-1	3,270E-1	7,950E-1	8,123E-1	9,757E-1
MAPK7	refseq_MAPK7.F4 refseq_MAPK7.R4	4,227E-1	9,953E-1	2,569E-1	9,788E-1	5,829E-1	6,229E-1	5,282E-1	8,451E-1	8,280E-1	9,620E-1	1,989E-1	9,916E-1	6,537E-1	8,166E-1	3,345E-1	9,005E-1
MEF2C	MEF2C.e01.F2 MEF2C.e01.R2	5,053E-1	4,861E-1	5,431E-1	9,923E-1	9,743E-1	9,764E-1	9,942E-1	7,421E-1	3,542E-1	6,435E-1	9,718E-1	9,151E-1	3,174E-1	8,641E-1	4,305E-1	7,584E-1
MEN1	refseq_MEN1.F1 refseq_MEN1.R1	7,314E-1	4,972E-1	2,408E-1	9,923E-1	6,934E-1	9,757E-1	5,328E-1	6,315E-1	5,395E-1	6,435E-1	3,585E-1	7,651E-1	8,756E-1	8,853E-1	2,519E-1	7,559E-1
METTL13	refseq_KIAA0859.F2 refseq_KIAA0859.R	2,195E-2	3,493E-1	2,379E-1	9,923E-1	5,449E-1	9,107E-1	4,404E-4	2,241E-2	4,097E-1	6,435E-1	1,099E-2	4,743E-1	7,744E-1	8,743E-1	3,916E-2	6,709E-1
MGRN1	MGRN1.m2h02.F1 MGRN1.m2h02.R1	7,507E-1	9,953E-1	4,918E-1	9,788E-1	4,533E-1	6,145E-1	8,243E-1	9,809E-1	8,258E-1	9,620E-1	4,568E-1	9,916E-1	5,833E-1	8,089E-1	9,514E-1	9,828E-1
MIER1	rs.MIER1.F1 rs.MIER1.R1	3,633E-1	4,786E-1	7,445E-1	9,923E-1	7,874E-1	9,757E-1	2,210E-1	4,805E-1	7,027E-1	6,507E-1	4,443E-1	7,651E-1	6,314E-1	8,743E-1	8,695E-1	8,932E-1
MITF	rs.MITF.F1 rs.MITF.R1	9,394E-1	9,953E-1	9,528E-1	9,788E-1	9,062E-1	7,304E-1	9,223E-1	9,884E-1	9,345E-1	9,968E-1	9,904E-1	9,916E-1	8,116E-1	8,498E-1	8,420E-1	9,757E-1
MLPH	rs.MLPH.F1 rs.MLPH.R1	6,484E-1	4,972E-1	2,521E-1	9,923E-1	5,309E-1	9,107E-1	6,329E-1	6,464E-1	7,108E-1	6,507E-1	1,677E-1	7,446E-1	8,571E-1	8,853E-1	3,901E-1	7,559E-1
MPZL1	refseq_MPZL1.F1 refseq_MPZL1.R1	9,427E-1	9,953E-1	6,513E-1	9,788E-1	8,259E-1	7,195E-1	5,711E-1	8,626E-1	7,651E-1	9,620E-1	7,586E-1	9,916E-1	6,658E-1	8,166E-1	4,671E-1	9,205E-1
MST4	rs.MST4.F1 rs.MST4.R1	4,152E-1	4,786E-1	4,305E-1	9,923E-1	5,621E-1	9,107E-1	6,659E-1	6,479E-1	9,796E-1	7,156E-1	3,805E-1	7,651E-1	5,288E-1	8,743E-1	6,214E-1	8,798E-1
MTDH	MTDH.m2h02.F1 MTDH.m2h02.R1	6,457E-1	4,972E-1	7,358E-1	9,923E-1	4,315E-1	9,107E-1	4,850E-1	6,224E-1	6,783E-1	6,435E-1	8,244E-1	9,121E-1	1,746E-1	7,016E-1	2,036E-1	7,559E-1
MTUS1	rs.MTUS1.F1 rs.MTUS1.R1	2,851E-1	4,786E-1	9,898E-1	9,940E-1	8,893E-1	9,764E-1	2,587E-1	5,076E-1	4,504E-1	6,435E-1	5,157E-1	8,243E-1	6,474E-1	8,743E-1	8,615E-1	8,932E-1
NBR1	refseq_NBR1.F1 refseq_NBR1.R1	9,826E-1	9,953E-1	5,552E-1	9,788E-1	6,104E-2	5,964E-1	1,260E-1	7,128E-1	7,071E-1	9,620E-1	4,957E-1	9,916E-1	1,243E-1	7,950E-1	2,516E-1	9,005E-1
NFATC2	refseq_NFATC2.F2 refseq_NFATC2.R2	1,624E-1	9,953E-1	4,958E-1	9,788E-1	5,131E-1	6,145E-1	3,920E-1	7,899E-1	6,294E-1	9,620E-1	1,829E-1	9,916E-1	3,190E-1	7,950E-1	7,578E-1	9,757E-1
NLRP1	DEFCAP.au.11.f NALP1.u.r.25	2,700E-1	9,953E-1	3,984E-1	9,788E-1	8,526E-1	7,195E-1	7,213E-1	9,617E-1	2,063E-1	9,620E-1	8,626E-1	9,916E-1	2,449E-1	7,950E-1	2,825E-1	9,005E-1
NLRP1	refseq_NALP1.F2 refseq_NALP1.R2	2,820E-1	9,953E-1	5,841E-1	9,788E-1	9,240E-1	7,304E-1	6,780E-1	9,617E-1	3,413E-1	9,620E-1	6,882E-1	9,916E-1	2,791E-1	7,950E-1	4,318E-1	9,205E-1
NLRP12	refseq_NALP12.F1 refseq_NALP12.R1	3,833E-1	4,786E-1	1,032E-2	7,349E-1	3,139E-2	4,126E-1	8,586E-1	7,104E-1	1,887E-1	6,435E-1	3,669E-2	6,332E-1	2,518E-1	8,172E-1	1,973E-1	7,559E-1
NLRP3	refseq_CIAS1.F1 refseq_CIAS1.R1	8,506E-1	5,392E-1	5,428E-1	9,923E-1	9,326E-2	6,675E-1	1,806E-1	4,805E-1	7,986E-1	6,606E-1	4,047E-1	7,651E-1	1,619E-1	6,830E-1	3,079E-1	7,559E-1
OAS1	refseq_OAS1.F1 refseq_OAS1.R1	8,841E-1	5,406E-1	5,678E-1	9,923E-1	7,925E-1	9,757E-1	9,700E-1	7,356E-1	6,404E-1	6,435E-1	7,790E-1	8,860E-1	8,211E-1	8,852E-1	6,366E-1	8,798E-1
OASL	refseq_OASL.F1 refseq_OASL.R1	2,122E-1	4,786E-1	5,854E-2	9,923E-1	3,220E-1	9,013E-1	1,911E-1	4,805E-1	5,595E-1	6,435E-1	8,110E-2	7,446E-1	7,560E-1	8,743E-1	1,105E-1	7,559E-1
OPA1	OPA1.m2h03.F1 OPA1.m2h03.R1	7,372E-1	4,972E-1	6,004E-1	9,923E-1	2,827E-1	8,969E-1	2,438E-1	5,010E-1	5,792E-1	6,435E-1	8,566E-1	9,130E-1	6,925E-2	5,666E-1	5,997E-2	6,709E-1
OPA1	OPA1.u.f.9 OPA1.u.r.8	5,345E-1	4,957E-1	4,628E-1	9,923E-1	5,964E-1	9,107E-1	5,570E-1	6,315E-1	4,271E-1	6,435E-1	9,139E-1	9,130E-1	6,340E-2	5,666E-1	6,078E-2	6,709E-1
OPA1	refseq_OPA1.F1 OPA1.u.r.6	5,403E-1	4,957E-1	4,818E-1	9,923E-1	8,918E-1	9,764E-1	7,963E-1	7,037E-1	4,135E-1	6,435E-1	9,455E-1	9,130E-1	3,913E-1	8,743E-1	2,944E-1	7,559E-1
OPA1	rs.OPA1.F2 rs.OPA1.R2	9,612E-1	5,523E-1	5,299E-1	9,923E-1	4,081E-1	9,107E-1	2,480E-1	5,010E-1	6,737E-1	6,435E-1	5,899E-1	8,485E-1	1,484E-1	6,830E-1	7,924E-2	6,709E-1
OPTN	rs.OPTN.F1 rs.OPTN.R1	3,015E-1	9,953E-1	4,281E-1	9,788E-1	7,638E-1	7,073E-1	4,250E-1	7,899E-1	2,739E-1	9,620E-1	3,933E-1	9,916E-1	4,723E-1	7,950E-1	4,901E-1	9,205E-1
OS9	refseq_OS9.F1 refseq_OS9.R1	9,953E-1	9,953E-1	9,603E-1	9,788E-1	4,917E-1	6,145E-1	4,674E-1	7,899E-1	9,791E-1	9,986E-1	9,635E-1	9,916E-1	3,501E-1	7,950E-1	3,200E-1	9,005E-1
PARL	refseq_PARL.F2 refseq_PARL.R2	8,930E-1	5,406E-1	3,075E-1	9,923E-1	6,614E-2	6,675E-1	2,015E-1	4,805E-1	5,103E-1	6,435E-1	3,745E-1	7,651E-1	6,501E-2	5,666E-1	2,754E-1	7,559E-1
PDCD4	refseq_PDCD4.F1 refseq_PDCD4.R1	3,666E-2	9,953E-1	2,489E-1	9,788E-1	8,478E-2	5,964E-1	5,670E-2	5,608E-1	4,590E-2	9,620E-1	4,703E-1	9,916E-1	3,556E-1	7,950E-1	8,591E-1	9,757E-1
PDPK1	refseq_PDPK1.F1 refseq_PDPK1.R1	2,388E-1	9,953E-1	6,942E-1	9,788E-1	8,324E-1	7,195E-1	3,848E-1	7,899E-1	5,615E-1	9,620E-1	3,494E-1	9,916E-1	4,908E-1	8,075E-1	7,452E-1	9,757E-1
PIDD	LRDD.u.f.7 LRDD.u.r.3	8,609E-1	5,393E-1	6,730E-1	9,923E-1	5,337E-1	9,107E-1	5,868E-1	6,315E-1	6,347E-1	6,435E-1	8,868E-1	9,130E-1	7,486E-1	8,743E-1	8,519E-1	8,932E-1
PIDD	refseq_LRDD.F4 refseq_LRDD.R4	5,879E-2	4,786E-1	6,075E-1	9,923E-1	5,062E-1	9,107E-1	4,664E-1	6,224E-1	4,642E-1	6,435E-1	2,394E-2	5,166E-1	4,527E-2	5,458E-1	9,611E-1	8,955E-1
PIDD	refseq_LRDD.F6 refseq_LRDD.R6	4,308E-1	4,786E-1	5,111E-1	9,923E-1	2,112E-2	4,077E-1	1,716E-3	2,947E-2	9,113E-1	6,989E-1	8,475E-2	7,446E-1	7,385E-2	5,666E-1	1,167E-2	4,941E-1
PILRA	refseq_PILRA.F1 refseq_PILRA.R1	8,492E-1	9,953E-1	1,611E-1	9,788E-1	1,376E-1	5,964E-1	7,594E-2	5,608E-1	2,717E-1	9,620E-1	4,282E-1	9,916E-1	6,124E-1	8,089E-1	8,421E-1	9,757E-1
PILRB	refseq_PILRB.F3 refseq_PILRB.R3	8,074E-1	9,953E-1	6,269E-1	9,788E-1	5,875E-1	6,229E-1	8,555E-1	9,809E-1	8,517E-1	9,620E-1	6,813E-1	9,916E-1	8,944E-1	8,789E-1	7,844E-1	9,757E-1
PORCN	refseq_PORCN.F1 refseq_PORCN.R1	2,662E-1	9,953E-1	4,214E-1	9,788E-1	4,980E-1	6,145E-1	4,578E-1	7,899E-1	3,009E-1	9,620E-1	7,805E-1	9,916E-1	4,652E-1	7,950E-1	6,792E-1	9,757E-1
PPARG	rs.PPARG.F1 rs.PPARG.R1	4,692E-1	9,953E-1	6,431E-1	9,788E-1	2,781E-1	5,964E-1	2,436E-1	7,899E-1	5,307E-1	9,620E-1	7,651E-1	9,916E-1	8,216E-1	8,498E-1	7,130E-1	9,757E-1
PRMT2	refseq_PRMT2.F2 refseq_PRMT2.R2	3,086E-1	4,786E-1	6,202E-1	9,923E-1	5,031E-1	9,107E-1	4,319E-2	2,966E-1	7,631E-1	6,587E-1	1,898E-1	7,446E-1	9,455E-1	8,853E-1	3,785E-1	7,559E-1
PTPN13	PTPN13.F7 PTPN13.R7	7,384E-1	4,972E-1	5,886E-1	9,923E-1	6,009E-1	9,107E-1	6,255E-1	6,464E-1	6,220E-1	6,435E-1	8,696E-1	9,130E-1	9,192E-1	8,853E-1	9,456E-1	8,955E-1
PTPN13	rs.PTPN13.F2 rs.PTPN13.R2	2,204E-1	4,786E-1	7,140E-1	9,923E-1	9,438E-1	9,764E-1	5,792E-1	6,315E-1	3,724E-1	6,435E-1	4,913E-1	8,000E-1	2,317E-1	8,171E-1	4,807E-1	7,981E-1
PYCARD	refseq_PYCARD.F1 refseq_PYCARD.R1	6,551E-1	9,953E-1	8,387E-1	9,788E-1	1,781E-1	5,964E-1	7,255E-2	5,608E-1	2,196E-1	9,620E-1	7,159E-1	9,916E-1	2,232E-1	7,950E-1	9,127E-2	8,888E-1
RAB3IP	refseq_RAB3IP.F1 refseq_RAB3IP.R1	3,597E-2	3,876E-1	9,273E-1	9,923E-1	1,758E-1	8,089E-1	1,504E-2	1,756E-1	8,988E-2	6,435E-1	1,846E-1	7,446E-1	7,531E-1	8,743E-1	2,757E-1	7,559E-1
RBCK1	refseq_RBCK1.F1 refseq_RBCK1.R1	6,242E-1	9,953E-1	8,755E-1	9,788E-1	7,710E-1	7,073E-1	9,020E-1	9,884E-1	7,224E-1	9,620E-1	7,666E-1	9,916E-1	3,738E-1	7,950E-1	4,188E-1	9,205E-1
RCAN1	DSCR1.e02.F1 DSCR1.e01.R1	7,062E-1	9,953E-1	3,984E-1	9,788E-1	1,892E-1	5,964E-1	2,752E-1	7,899E-1	6,404E-1	9,620E-1	4,500E-1	9,916E-1	3,310E-1	7,950E-1	6,066E-1	9,757E-1
RHOT1	refseq_RHOT1.F1 refseq_RHOT1.R1	3,979E-1	4,786E-1	8,669E-1	9,923E-1	1,471E-1	7,253E-1	3,486E-2	2,660E-1	3,987E-1	6,435E-1	6,473E-1	8,768E-1	4,548E-1	8,743E-1	1,878E-1	7,559E-1
DSTYK	refseq_RIPK5.F1 refseq_RIPK5.R1	9,498E-1	9,953E-1	4,699E-1	9,788E-1	3,850E-1	6,145E-1	5,751E-1	8,626E-1	6,159E-1	9,620E-1	6,468E-1	9,916E-1	5,857E-1	8,089E-1	9,249E-1	9,757E-1
RNF138	refseq_RNF138.F1 refseq_RNF138.R1	5,099E-1	9,953E-1	9,557E-1	9,788E-1	5,352E-1	6,194E-1	1,499E-1	7,128E-1	7,014E-1	9,620E-1	6,135E-1	9,916E-1	8,558E-1	8,747E-1	4,945E-1	9,205E-1
RREB1	refseq_RREB1.F1 refseq_RREB1.R1	4,341E-1	4,786E-1	1,986E-1	9,923E-1	1,498E-1	7,253E-1	2,144E-1	4,805E-1	9,190E-2	6,435E-1	7,687E-1	8,860E-1	8,083E-1	8,852E-1	8,976E-1	8,932E-1
RUNX2	RUNX2.F1 RUNX2.R1	9,427E-1	9,953E-1	2,146E-1	9,788E-1	1,809E-1	5,964E-1	6,281E-1	9,136E-1	4,928E-1	9,620E-1	2,969E-1	9,916E-1	5,444E-1	8,089E-1	7,740E-1	9,757E-1
RUNX2	rs.RUNX2.F1 rs.RUNX2.R1	4,438E-1	9,953E-1	5,543E-1	9,788E-1	6,044E-1	6,229E-1	6,946E-1	9,617E-1	9,148E-1	9,867E-1	3,512E-1	9,916E-1	5,018E-1	8,075E-1	7,640E-1	9,757E-1
SIRT2	SIRT2.e25.F1 SIRT2.e25.R1	8,189E-1	5,252E-1	8,830E-1	9,923E-1	6,007E-1	9,107E-1	8,276E-1	7,037E-1	9,599E-1	7,156E-1	7,905E-1	8,860E-1	6,821E-1	8,743E-1	8,434E-1	8,932E-1
SLA2	refseq_SLA2.F1 refseq_SLA2.R1	3,392E-1	4,786E-1	8,260E-1	9,923E-1	2,263E-1	8,969E-1	9,281E-1	7,326E-1	5,668E-1	6,435E-1	2,005E-1	7,525E-1	3,754E-1	8,743E-1	5,861E-1	8,798E-1
SNAP25	SNAP25.e07.F1 SNAP25.e07.R1	7,869E-1	5,108E-1	6,991E-1	9,923E-1	9,636E-1	9,764E-1	9,748E-1	7,356E-1	6,567E-1	6,435E-1	9,408E-1	9,130E-1	7,091E-1	8,743E-1	6,427E-1	8,798E-1
SPINT1	refseq_SPINT1.F1 refseq_SPINT1.R1	8,788E-1	5,406E-1	5,313E-1	9,923E-1	7,642E-2	6,675E-1	2,239E-1	4,805E-1	5,890E-1	6,435E-1	7,287E-1	8,860E-1	2,862E-1	8,366E-1	5,155E-1	8,235E-1
SPP1	SPP1-3 SPP1-4	7,475E-1	4,972E-1	9,618E-1	9,923E-1	5,816E-1	9,107E-1	6,836E-1	6,520E-1	8,750E-1	6,986E-1	7,567E-1	8,860E-1	4,037E-1	8,743E-1	4,389E-1	7,584E-1
SPP1	rs.SPP1.F2 rs.SPP1.R2	2,488E-1	4,786E-1	8,782E-1	9,923E-1	4,395E-1	9,107E-1	1,335E-1	4,672E-1	5,634E-1	6,435E-1	2,185E-1	7,542E-1	7,874E-1	8,743E-1	1,482E-1	7,559E-1
STAT3	rs.STAT3.F2 rs.STAT3.R2	6,177E-1	9,953E-1	8,355E-1	9,788E-1	5,952E-1	6,229E-1	9,115E-1	9,884E-1	6,397E-1	9,620E-1	8,482E-1	9,916E-1	4,545E-1	7,950E-1	6,520E-1	9,757E-1
SUCO	refseq_C1orf9.F2 refseq_C1orf9.R2	4,840E-1	4,861E-1	9,661E-1	9,923E-1	2,581E-1	8,969E-1	1,253E-1	4,672E-1	6,442E-1	6,435E-1	5,689E-1	8,464E-1	4,889E-1	8,743E-1	2,481E-1	7,559E-1
TBC1D25	refseq_OATL1.F1 refseq_OATL1.R1	2,593E-2	3,493E-1	6,892E-1	9,923E-1	4,281E-1	9,107E-1	3,339E-1	5,827E-1	1,566E-1	6,435E-1	1,265E-1	7,446E-1	3,241E-2	5,220E-1	9,072E-1	8,932E-1
TCF4	rs.TCF4.F1 rs.TCF4.R1	3,248E-1	9,953E-1	9,164E-1	9,788E-1	4,253E-1	6,145E-1	1,912E-1	7,851E-1	6,068E-1	9,620E-1	3,510E-1	9,916E-1	8,180E-1	8,498E-1	3,639E-1	9,005E-1
TCF7L2	TCF7L2.e01.F1 TCF7L2.e01.R1	7,982E-1	9,953E-1	6,601E-1	9,788E-1	3,620E-1	6,145E-1	4,191E-1	7,899E-1	7,185E-1	9,620E-1	8,150E-1	9,916E-1	5,572E-1	8,089E-1	6,469E-1	9,757E-1
TCF7L2	TCF7L2.e03.F2 TCF7L2.e03.R2	4,137E-1	9,953E-1	6,994E-1	9,788E-1	9,993E-1	7,590E-1	4,690E-1	7,899E-1	8,102E-1	9,620E-1	3,147E-1	9,916E-1	7,577E-1	8,448E-1	5,070E-1	9,205E-1
TCF7L2	TCF7L2.e04.F1 TCF7L2.e04.R1	6,112E-1	9,953E-1	8,959E-1	9,788E-1	6,198E-1	6,229E-1	9,539E-1	9,884E-1	8,284E-1	9,620E-1	6,101E-1	9,916E-1	3,980E-1	7,950E-1	7,601E-1	9,757E-1
TCOF1	refseq_TCOF1.F2 refseq_TCOF1.R2	4,174E-1	4,786E-1	3,503E-1	9,923E-1	3,836E-1	9,107E-1	4,548E-1	6,224E-1	3,314E-1	6,435E-1	9,142E-1	9,130E-1	7,838E-1	8,743E-1	7,415E-1	8,932E-1
TCTN3	refseq_C10orf61.F2 refseq_C10orf61.R2	2,682E-1	4,786E-1	7,883E-1	9,923E-1	2,990E-1	9,013E-1	3,765E-1	5,877E-1	1,328E-1	6,435E-1	6,658E-1	8,768E-1	6,150E-1	8,743E-1	8,498E-1	8,932E-1
TFEC	refseq_TFEC.F2 refseq_TFEC.R2	4,454E-1	9,953E-1	9,646E-1	9,788E-1	7,536E-1	7,073E-1	8,430E-1	9,809E-1	6,519E-1	9,620E-1	5,464E-1	9,916E-1	4,257E-1	7,950E-1	7,670E-1	9,757E-1
THAP1	refseq_THAP1.F1 refseq_THAP1.R1	6,229E-2	4,786E-1	8,104E-1	9,923E-1	9,209E-1	9,764E-1	3,186E-2	2,660E-1	5,571E-3	1,941E-1	4,234E-1	7,651E-1	3,426E-1	8,743E-1	6,872E-1	8,798E-1
THYN1	refseq_THYN1.F1 refseq_THYN1.R1	6,728E-1	9,953E-1	6,706E-1	9,788E-1	3,683E-1	6,145E-1	6,890E-2	5,608E-1	9,986E-1	9,986E-1	5,688E-1	9,916E-1	3,246E-1	7,950E-1	1,637E-2	5,237E-1
TM2D2	refseq_TM2D2.F3 refseq_TM2D2.R3	9,044E-1	5,414E-1	1,017E-1	9,923E-1	4,710E-1	9,107E-1	6,223E-1	6,464E-1	2,722E-1	6,435E-1	2,836E-1	7,651E-1	6,192E-1	8,743E-1	1,719E-1	7,559E-1
TNFRSF10B	TNFRSF10B.u.f.10 tnfrsf10b.r.3	1,621E-1	9,953E-1	7,403E-1	9,788E-1	6,169E-2	5,964E-1	3,329E-2	5,608E-1	2,626E-1	9,620E-1	3,716E-1	9,916E-1	7,528E-1	8,448E-1	3,562E-1	9,005E-1
TPD52L1	refseq_TPD52L1.F1 refseq_TPD52L1.R1	4,815E-1	4,861E-1	7,664E-1	9,923E-1	5,727E-1	9,107E-1	4,771E-1	6,224E-1	6,329E-1	6,435E-1	6,322E-1	8,768E-1	9,337E-1	8,853E-1	7,312E-1	8,932E-1
TPD52L2	rs.TPD52L2.F1 rs.TPD52L2.R1	5,705E-1	4,957E-1	5,216E-1	9,923E-1	3,050E-1	9,013E-1	7,622E-1	7,037E-1	9,693E-1	7,156E-1	1,721E-1	7,446E-1	3,091E-1	8,641E-1	7,921E-1	8,932E-1
TRAF3	TRAF3.u.f.1 TRAF3.u.r.3																
TRAF3	rs.TRAF3.F1 rs.TRAF3.R1																
TRPC4AP	refseq_TRPC4AP.F1 refseq_TRPC4AP.R1	8,995E-1	9,953E-1	7,542E-1	9,788E-1	5,770E-1	6,229E-1	4,614E-1	7,899E-1	9,088E-1	9,867E-1	7,309E-1	9,916E-1	2,332E-1	7,950E-1	2,289E-1	9,005E-1
TSSC4	TSSC4.F10 TSSC4.R7	4,353E-1	4,786E-1	5,934E-1	9,923E-1	3,331E-1	9,013E-1	9,111E-1	7,275E-1	8,682E-1	6,986E-1	3,967E-1	7,651E-1	3,282E-1	8,654E-1	9,703E-1	8,955E-1
UBE2D3	refseq_UBE2D3.F2 refseq_UBE2D3.R2	7,872E-1	9,953E-1	5,031E-1	9,788E-1	4,068E-1	6,145E-1	7,869E-1	9,809E-1	7,807E-1	9,620E-1	5,509E-1	9,916E-1	5,514E-1	8,089E-1	9,954E-1	9,954E-1
UBE2V1	refseq_UBE2V1.F1 refseq_UBE2V1.R1	9,528E-2	9,953E-1	7,808E-1	9,788E-1	3,717E-1	6,145E-1	9,942E-1	9,942E-1	1,715E-1	9,620E-1	3,236E-1	9,916E-1	1,991E-2	7,505E-1	1,246E-1	9,005E-1
USP4	refseq_USP4.F1 refseq_USP4.R1	1,923E-1	4,786E-1	2,399E-1	9,923E-1	1,318E-2	4,077E-1	1,032E-3	2,363E-2	8,751E-2	6,435E-1	9,063E-1	9,130E-1	5,026E-1	8,743E-1	3,933E-1	7,559E-1
VHL	rs.VHL.F1 rs.VHL.R1	9,027E-1	9,953E-1	8,229E-1	9,788E-1	9,256E-1	7,304E-1	8,682E-1	9,809E-1	9,513E-1	9,986E-1	8,065E-1	9,916E-1	9,984E-1	8,929E-1	8,530E-1	9,757E-1
WARS	refseq_WARS.F1 refseq_WARS.R1	6,753E-1	4,972E-1	3,814E-1	9,923E-1	9,457E-1	9,764E-1	4,899E-1	6,224E-1	6,842E-1	6,435E-1	4,792E-1	7,954E-1	7,375E-1	8,743E-1	3,889E-1	7,559E-1
WARS	refseq_WARS.F3 refseq_WARS.R3	7,939E-2	4,786E-1	9,712E-1	9,923E-1	3,331E-1	9,013E-1	7,996E-1	7,037E-1	1,215E-1	6,435E-1	3,182E-1	7,651E-1	2,313E-2	5,220E-1	3,714E-1	7,559E-1
ZDHHC13	refseq_ZDHHC13.F1 refseq_ZDHHC13.R1	7,218E-1	4,972E-1	3,360E-1	9,923E-1	7,460E-1	9,757E-1	6,494E-1	6,464E-1	6,749E-1	6,435E-1	4,073E-1	7,651E-1	9,344E-1	8,853E-1	3,366E-1	7,559E-1
ZDHHC16	refseq_ZDHHC16.F2 refseq_ZDHHC16.R2	7,212E-1	4,972E-1	7,805E-2	9,923E-1	2,688E-1	8,969E-1	8,907E-1	7,196E-1	1,601E-1	6,435E-1	3,619E-1	7,651E-1	7,078E-1	8,743E-1	2,256E-1	7,559E-1
ZDHHC16	refseq_ZDHHC16.F6 refseq_ZDHHC16.R6	3,003E-1	4,786E-1	9,263E-1	9,923E-1	3,920E-1	9,107E-1	8,301E-1	7,037E-1	5,289E-1	6,435E-1	3,347E-1	7,651E-1	1,260E-1	6,730E-1	3,363E-1	7,559E-1
